# An analytical pipeline for identifying and mapping the integration sites of HIV and other retroviruses

**DOI:** 10.1186/s12864-020-6647-4

**Published:** 2020-03-09

**Authors:** Daria W. Wells, Shuang Guo, Wei Shao, Michael J. Bale, John M. Coffin, Stephen H. Hughes, Xiaolin Wu

**Affiliations:** 1 Cancer Research Technology Program, Leidos Biomedical Research, Inc., Frederick National Laboratory for Cancer Research, PO Box B, Frederick, MD 21702 USA; 2 Advanced Biomedical Computational Science, Leidos Biomedical Research, Inc., Frederick National Laboratory for Cancer Research, Frederick, MD USA; 3 HIV Dynamics and Replication Program, National Cancer Institute Frederick, National Institutes of Health, Frederick, MD USA; 4 Department of Molecular Biology and Microbiology, Tufts University, Boston, MA USA

**Keywords:** Retrovirus, Patient samples, HIV, Integration, PCR mispriming, PCR recombination

## Abstract

**Background:**

All retroviruses, including human immunodeficiency virus (HIV), must integrate a DNA copy of their genomes into the genome of the infected host cell to replicate. Although integrated retroviral DNA, known as a provirus, can be found at many sites in the host genome, integration is not random. The adaption of linker-mediated PCR (LM-PCR) protocols for high-throughput integration site mapping, using randomly-sheared genomic DNA and Illumina paired-end sequencing, has dramatically increased the number of mapped integration sites. Analysis of samples from human donors has shown that there is clonal expansion of HIV infected cells and that clonal expansion makes an important contribution to HIV persistence. However, analysis of HIV integration sites in samples taken from patients requires extensive PCR amplification and high-throughput sequencing, which makes the methodology prone to certain specific artifacts.

**Results:**

To address the problems with artifacts, we use a comprehensive approach involving experimental procedures linked to a bioinformatics analysis pipeline. Using this combined approach, we are able to reduce the number of PCR/sequencing artifacts that arise and identify the ones that remain. Our streamlined workflow combines random cleavage of the DNA in the samples, end repair, and linker ligation in a single step. We provide guidance on primer and linker design that reduces some of the common artifacts. We also discuss how to identify and remove some of the common artifacts, including the products of PCR mispriming and PCR recombination, that have appeared in some published studies. Our improved bioinformatics pipeline rapidly parses the sequencing data and identifies bona fide integration sites in clonally expanded cells, producing an Excel-formatted report that can be used for additional data processing.

**Conclusions:**

We provide a detailed protocol that reduces the prevalence of artifacts that arise in the analysis of retroviral integration site data generated from in vivo samples and a bioinformatics pipeline that is able to remove the artifacts that remain.

## Background

Integration of a DNA copy of the viral genome into the host genome, forming a provirus, is an essential step in the replication of all retroviruses. However, the overall distribution of integration sites differs for different types of retroviruses. For example, primate lentiviruses, including HIV-1, preferentially integrate into the bodies of expressed genes in gene-dense regions [1]. This distribution is a consequence of interactions of the integration machinery with specific cellular factors, including CPSF6 and p75 LEDGF [2, 3]. By contrast, the integration machinery of murine leukemia virus (MLV) interacts with a different set of host factors, including the BET proteins [4–6], and MLV proviruses preferentially integrate near active enhancers [7–9]. Integration in specific regions in or near oncogenes can lead to clonal expansion of the infected cells, and, in some cases, to the development of tumors [10]. Analysis of integration sites has been used to track the clonal expansion of HTLV and HIV infected cells in patients [11–13], and to follow the behavior of cells that have been modified by retroviral vectors in gene therapy patients [14–19].

In HIV infected people, the majority of infected cells are CD4+ T cells. Some of the infected T cells proliferate and grow into clones of infected cells that can persist for more than 10 years during effective anti-retroviral therapy (ART) [12, 13]. Current ART effectively blocks viral replication but does not eliminate persistently infected cells. In patients on long-term ART, most of the infected cells carry defective proviruses [20]. Despite contradictory claims in the literature [21], it is now clear that clonally expanded cells can carry an intact infectious provirus and can release low levels of infectious virus into the blood [22]. It appears that the majority of the proviruses in clones of expanded cells are transcriptionally silent [23] explaining how some infected cells can survive and expand into clones. However, at least some of the dormant proviruses in the cells that make up these clones can be activated to express infectious virus. This reactivation leads to a rapid rekindling of the infection if ART is interrupted [24, 25].

All of the descendants of an infected cell will inherit a copy of a provirus integrated at exactly the same specific site in the host genome as in the parental cell. In samples obtained from patients, integration sites can be used to track clonal expansion and persistence of infected cells and integration site data can be used to identify proviruses that affect the growth and survival of the infected cells. Linker-mediated PCR (LM-PCR) has been widely used for integration site analysis [1, 8, 11]. A DNA linker is ligated onto the ends of the fragmented DNA followed by selective PCR amplification of host/virus DNA junctions using primers specific for viral DNA and for the ligated linker. In the initial versions of the protocol, used by the Bushman lab [1] and the Burgess lab [8], genomic DNA containing integrated proviruses was fragmented by digestion with various restriction enzymes. A double stranded DNA-linker was ligated to the resulting DNA fragment and the amplified products were analyzed by Sanger sequencing [1, 8]. The non-uniform distribution of restriction sites in the host genome meant that some integration sites were preferentially amplified and sequenced and some integration sites were missed if there were no suitable restriction sites nearby. Sanger sequencing also limited the number of integration sites that could be analyzed. Fragmenting the host DNA using sonication instead of restriction enzyme digestion and replacing Sanger sequencing with deep sequencing methods (initially 454, more recently Illumina) made it possible to identify many more integration sites. The Bangham lab improved integration site analysis using randomly sheared DNA and paired-end Illumina sequencing [11], making it possible to detect the presence of multiple copies of the same integration site that originated from the clonal expansion of infected cells. Adding a single dA to the ends of the sheared DNA, coupled with the use of a linker with a corresponding one nucleotide dT overhang dramatically reduced unwanted non-specific linker-linker and genomic-genomic DNA ligation [11]. The protocol can be used to analyze the integration sites of all types of retroviruses. However, significant optimization is needed depending on the samples that are being analyzed. For example, in the analysis of samples from gene therapy patients treated with a retroviral vector, accurate quantitation of the sizes of the clones is usually desirable. However, for studies involving samples from HIV-infected patients, sensitivity and the elimination of artifacts are the major concerns.

In cultured cells, it is relatively easy to infect most or all of the cells with HIV, leading to abundant proviruses, and it is relatively easy to perform integration site analysis. We have identified millions of integration sites from cells infected in culture. However, in HIV infected individuals, only a small fraction of the CD4+ T cells, often less than one in a thousand, are infected. This scarcity of proviruses poses significant challenges for the method. To identify HIV integration sites in samples taken from patients, PCR amplification must be both efficient and highly specific. Even with the best of the available techniques, a large fraction of the DNA sequences that are obtained do not correspond to valid integration sites due to PCR mispriming, PCR recombination, and other artifacts (discussed below and in the Supplementary information). In some samples, the majority of the amplified DNA sequences are unwanted non-specific PCR products. Because vast amounts of data are obtained by deep sequencing methods, computer programs/algorithms must be used to identify valid integration sites and discard any unwanted sequences. Here we present improved and optimized methods for integration site analysis using linker-mediated PCR for library construction and a bioinformatics pipeline that parses the resulting Illumina sequencing data from HIV patient samples. The pipeline identifies and maps valid integration sites and is designed to recognize and eliminate common artifacts that arise when only a small fraction of the cells in the starting samples contain a provirus of interest. The pipeline can also be used to identify clonally expanded cells and to estimate the relative size of each clone.

## Results

The protocol we describe here has been optimized to amplify the relatively rare HIV integration sites present in samples taken from patients. We present both a selective amplification protocol designed to reduce the artifacts commonly arising during linker-mediated PCR protocols that involve high levels of amplification and a matching bioinformatics pipeline designed to remove the residual artifacts. We have included an overview of an analysis of 3 clinical samples from HIV infected individuals that were done using the experimental procedures we describe and the bioinformatics pipeline. The samples were obtained from patients who were on long-term combination antiretroviral therapy.

### Selective amplification of integration sites

**Fig. 1:**
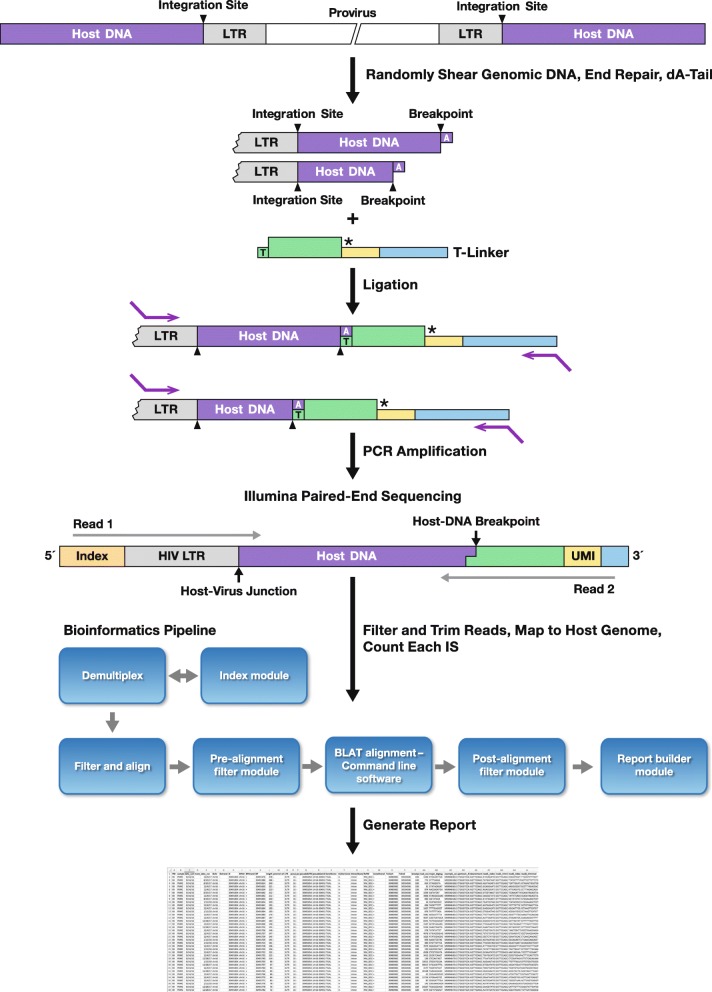
Overview of the linker-mediated amplification of HIV integration sites and the bioinformatic pipeline used to distinguish real integration sites from artifacts. A schematic drawing of an integrated HIV provirus is shown at the top of the figure. As described in the text, and in the Supplementary material, the host DNA is fragmented, the ends are made blunt, and a single dA is added to the 3′ ends of the fragmented host DNA. The T-linker is composed of a long and a short oligonucleotide. The 3′ end of the short oligonucleotide is blocked (marked by an asterisk, see text) to prevent it from being extended in the PCR steps (see Figs. 2 and 3 for the design and sequences of the linker and primers). The UMI, which is shown in yellow, is a random sequence that is used to help determine if two similar amplified segments do (or do not) originate from two different starting pieces of host DNA. In the example shown in the figure (middle of the drawing), next to T-linkers, there are two fragments that contain a host-virus DNA junction that come from a clone of expanded cells. The two fragments have exactly the same integration site, but there are different breakpoints in the host DNA. As described in the text, the PCR reaction must be initiated from the LTR primer because the segment that the first T-linker primer anneals to is not present in the short oligonucleotide of the T-linker. The bottom of the drawing shows a schematic diagram of the amplified DNA and the location of the Illumina Read 1 and Read 2 primers. The Illumina data are processed, using the pipeline, as indicated in the diagram, and the output is saved as an Excel file (the screen shot shows a small portion of an output file)

Integration sites are amplified using a linker-mediated PCR protocol with modifications to improve the specificity of the amplification and reduce artifacts. The overall workflow used in construction of the integration site libraries is shown in Fig. 1. We selectively amplify the host/provirus DNA junctions from both the 3′ and the 5′ LTRs. Simultaneous amplification of both the 3′ and 5′ LTR junctions increases the sensitivity of detection of valid integration sites and the confidence that the identified sites are valid. Libraries are generated from genomic DNA from HIV-infected cells using the NEBNext Ultra II FS DNA Library Prep Kit for Illumina with some modifications. The Ultra II FS Kit combines several of the initial DNA preparation steps including 1) DNA fragmentation, 2) end-repair, 3) linker-ligation into a single step. The kit replaces the original mechanical shearing of DNA with enzymatic shearing using a dsDNA fragmentase designed specifically to produce fragments that are appropriate for next generation sequencing. This enzymatic approach results in random fragmentation and avoids the use of an expensive Covaris instrument, which is needed if the fragments are produced by acoustical shearing. The enzymatic approach can be performed in parallel with multiple samples. Enzymatic shearing helps to preserve limited amounts of DNA present in precious samples. The new protocol uses only 1 μg of the DNA input comparing to 3 μg in our previous protocol in which acoustical shearing was used. The protocol can be performed with little as 50 ng of input DNA. We use an accurate digital droplet PCR (ddPCR) protocol to determine the number of HIV proviruses that are present in the samples. The procedures we use do not recover all of the integration sites that are present in the sample. The shearing procedure and the manipulation of the samples leads to a loss of some of the starting DNA and the PCR amplification of the fragments that contain the host virus DNA junctions is not 100%. We usually recover 5–15% of the integration sites that were present in the input DNA. We recently modified our procedure to introduce a unique molecular identifier (UMI) sequence tag (8 or more random nucleotides) in the single stranded portion of the partially double stranded T-linker (Fig. 1). After linker ligation, each of the randomly sheared DNA fragments that contains an integration site will carry a UMI, which is amplified and sequenced with the integration site. The UMI helps to solve one of the limitations of using sheared ends to distinguish independent DNA molecules with the same integration site. When the number of expanded cells in a clone is large, a sample can contain hundreds of independent DNA molecules that have the same integration site. Because the fragments are only a few hundred nucleotides long, some will have the same or similar sheared ends. If, in such cases, only the unique sheared ends are counted, the number of different molecules with the same integration site is undercounted. In addition, amplification and sequencing errors can lead to the generation, from a single starting DNA fragment, of a family of reads with closely related breakpoints (which we refer to as “fuzz”). When this happens, UMIs can be used to show that identical integration sites are (or are not) derived from same or independent DNA molecules. However, as will be discussed in more detail below, there are also artifacts associated with the use of UMIs. Thus, to accurately count the number of molecules with the same integration site, the best approach is to use breakpoints in combination with UMIs.

### T-linker-UMI and the PCR primers

#### T-linker-UMI

**Fig. 2:**
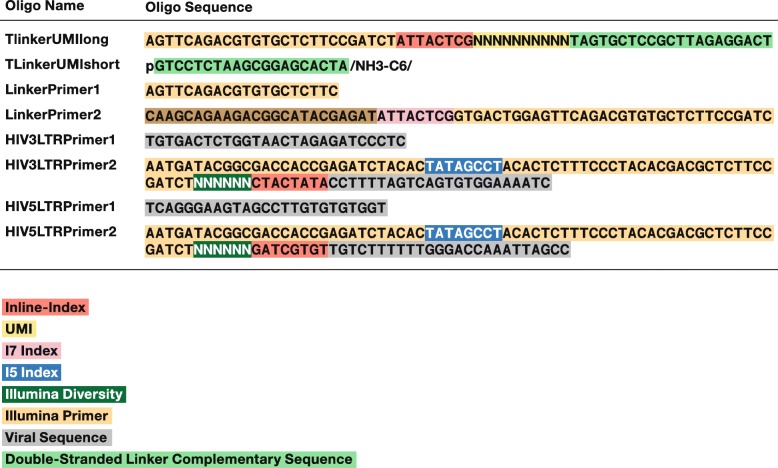
The sequences of the primers and adaptors used in the selective amplification of the host-virus junctions. The segments of the oligonucleotides are color coded in the figure; the key to the color coding is shown in the figure

The structure of the T-Linker-UMI, which is ligated to the ends of the sheared DNA, is shown in Fig. 1, and its sequence is given in Fig. 2. The shorter strand is an oligonucleotide with the 5′ end phosphorylated (to enable efficient linker ligation) and the 3′ end modified with hexanediol, a six-carbon glycol, which blocks extension of the short strand by DNA polymerase. The longer strand includes a 3′-T overhang, a sequence complimentary to the corresponding short strand, a UMI that is 8-nucleotides-long or longer, and the Illumina PE2 SP adapter sequence. The UMI portion of the linker is synthesized using a mixture of equal amounts of dA, dT, dC and dG (shown as Ns) at each position to create diverse sequences (Figs. 1 and 2).

#### First PCR primer

The first PCR reaction is initiated using primers that are designed to match sequences near the ends of each LTR. The primers are typically around 20–25 nucleotides long. Because there is considerable variation in the sequence of the HIV proviruses in patients, we usually determine the sequences of the ends of the LTRs for the proviruses in each patient and prepare specific primers to match the proviruses present, which usually improve the recovery of the integration sites. A “generic” primer matching conserved regions can be used in the PCR amplification from patient samples. Because the U3 region of the HIV LTR is more conserved than the U5 region, in most cases a U3 “generic” primer will perform better than the U5 “generic” primer with clinical samples. To prepare patient specific primers, we determine the sequence of the LTRs of the proviruses in the patient. To avoid unwanted amplification of host DNA, the first primer that will amplify from the T-linker matches the single-stranded portion of the 5′ end of the long strand (Fig. 2). Thus, the first T-linker primer has no target to which it can anneal until a DNA strand has been synthesized, starting from the viral primer, that includes a copy of the single stranded portion of the adaptor. The 3′ C6 modification of the short strand of the linker prevents its extension by the DNA polymerase, which helps prevent the generation of false products from the linker primer. In the most recent version of the protocol, we usually use 1 μg amounts of DNA in the amplification reactions. This is equivalent to the amount of DNA in about 165,000 cells. Because the number of proviruses in patient samples is low, 1 μg may not yield the desired number of integration sites. If more integration sites are needed, multiple reactions are run in parallel.

#### Nested PCR

**Fig. 3:**
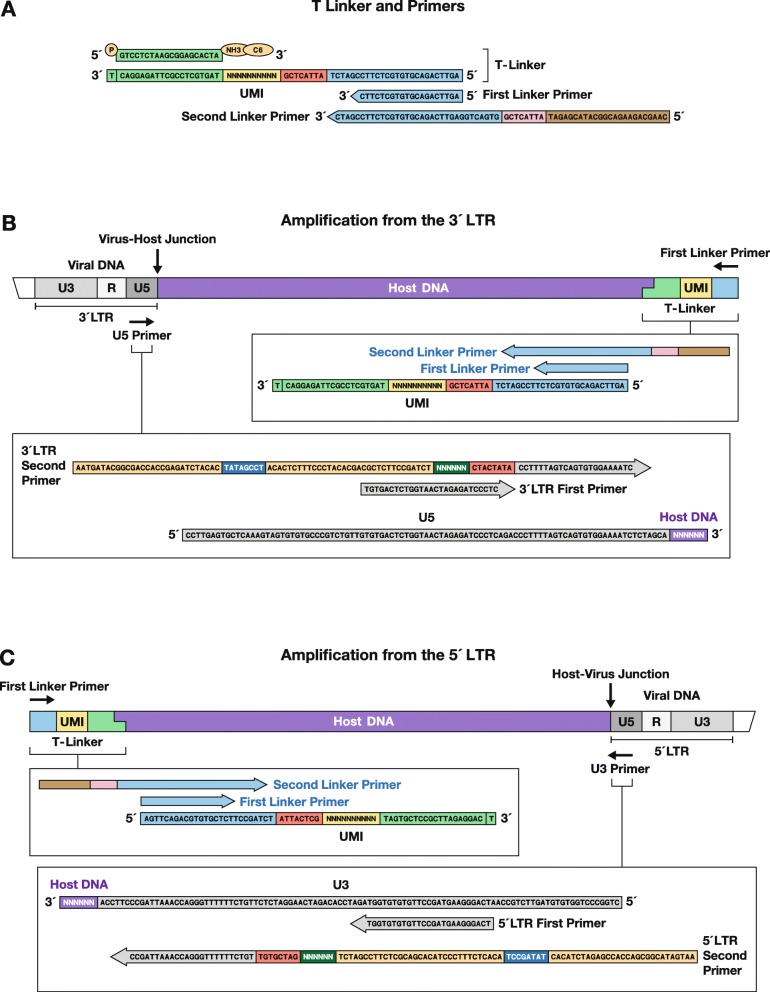
PCR primers used in the amplification reaction, and their cognate substrates. We amplify both LTR/host junctions and the primers for both ends of the provirus are shown. Panel **a**. At the top is a drawing of the T-linker, and the first and second linker primers. The blocking group (NH3, C6) on the 3′ end of the short strand of the T-linker is shown, and the 5′ end of short strand is phosphorylated (P), to allow it to be ligated to the host DNA. The second linker primer is used to add the sequences needed for Illumina sequencing to one end of the amplified fragment. The first and second LTR primers used to amplify the 5′ LTR are shown in the drawing, with the linker primers used to amplify the host-virus DNA junction. The sequences needed for Illumina sequencing to the other end of the amplified fragments are added as part of the second LTR primer. Panel **b** shows the amplification of the 3′ LTR and the appended host sequences, together with the first and second LTR primers (see also Fig. 2). Panel **c** At the bottom of the drawing, are the corresponding primers used to amplify the 5′ LTR

Although the first round of PCR amplification is designed to selectively amplify the LTR/host DNA junctions, single round amplification does not generate enough desired products for Illumina sequencing. Nested PCR is used to increase the yield of LTR/host junction products. Nested PCR primers match sequences inside (3′ of) the first round PCR primers and are used to increase both the yield and the specificity of the amplification. Illumina adaptors and an inline index (barcode) are added during the nested PCR step. Primers for the LTR end of the amplicon include an Illumina P5 grafting sequence, an 8-nucleotide i5 index, an Illumina PE1 SP adapter sequence, a 10-nucleotide UMI, an 8-nucleotide inline index, and a 20–25 nucleotide LTR specific sequence (Fig. 2). Primers for the linker end are composed of an Illumina P7 grafting sequence, an 8-nucleotide i7 index and Illumina PE2 SP sequence (Figs. 2 and 3). The i5 and i7 indexes are used by the sequencer for initial sorting during fastq file generation and inline indexes are used for secondary sorting during the de-multiplexing step in our pipeline.

### Bioinformatic analysis

**Fig. 4:**
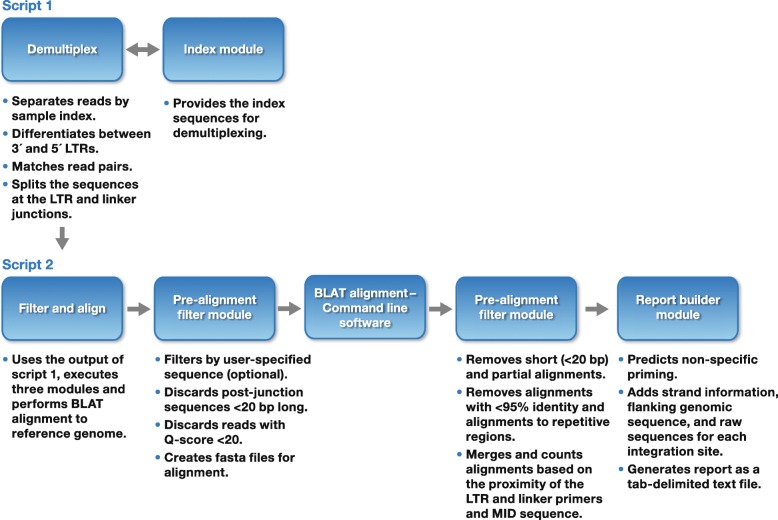
The bioinformatic pipeline. The boxes show how the pipeline processes the Illumina data. For a more detailed explanation, please see the main text

The Illumina sequencing data are analyzed using our custom pipeline, which is illustrated in the flowchart shown in Fig. 4. The pipeline consists of several major steps: 1) Sample demultiplexing. 2) Pre-alignment trimming and filtering. 3) Genome alignment. 4) Post-alignment filtering and removal of artifacts. 5) Annotation and report generation. 6) Re-analyzing unmappable reads. The pipeline is written in Perl and uses BLAT (a BLAST-like alignment tool) for genome alignment. It is very efficient and can be installed on a Mac (laptop or desktop) or on a Unix server. The execution times under various computational settings/OS for typical datasets are listed in Supplementary Table 1. Most of our processing is done in Mac OSX using a Mac laptop with single thread processing. Each step is discussed below.

#### Sample demultiplexing

Illumina sequencing produces millions of reads for each run. Multiple samples, marked with different inline index sequences, can be sequenced in the same run, reducing the cost and increasing the number of samples that are analyzed. After the run, paired-end sequence reads are demultiplexed and assigned to each sample based on the Illumina sequencing index (these are the i5 and i7 indexes, which are not the same as the inline indexes, see Fig. 2). The demultiplexing can be done using Illumina software. During the demultiplexing process, we separate the reads that are derived from the 3’LTR junctions and the 5’LTR junctions based on the primer sequences, which are detected in read1. However, there are problems that are known to occur in multiplex-sequencing, for example “index hopping” in which reads that belong to one sample are assigned to another. Misassignment can happen if there are sequencing errors in the inline index or contamination with a different inline index in the PCR step. As noted earlier, we have found that primers with different inline indexes can be cross-contaminated if they are ordered from a single supplier at the same time. We do not include multiple primers in the same order from a single supplier. Even though these events are relatively rare, they can cause problems in experiments in which the goal is to identify rare integration sites. Care must be taken to reduce the number of such events and identify them if they occur. Although we initially used only a single inline index, we recently switched to using two inline indexes. The inline indexes are included as part of both the read1 and read2 sequences. Our bioinformatics pipeline checks the sequences of the inline indexes. This protocol prevents mis-assignment of reads even if one of the outside Illumina indexes has a sequencing error.

The sample names, indexes sequences, and related information are stored in a plain text settings file. The first script reads the settings file and scans the fastq files, in gzip format, that were generated by Illumina sequencer and demultiplexes the data into separate files for each sample.

#### Pre-alignment filtering and trimming

This step is very important for precise mapping of integration sites and breakpoints in the genome. It is used to remove the majority of the reads that do not correspond to integration sites.

Demultiplexed read1 and read2 sequences from each sample are processed in pairs. 3’LTR and 5’LTR junction sequences are processed separately because the LTR primer sequences are different. The LTR nested primers are designed to initiate DNA synthesis close to the end of the LTR but at least a few nucleotides away from the end so that mispriming events can be identified (see Discussion). For the read1 sequence, the script first looks for the sequence of the nested primer that matches the HIV LTR and checks for the expected sequence from the end of the primer to the end of the LTR (typically 5 or more nucleotides).

For read2 sequences, the script first looks for the sequence of the linker nested PCR primers, then extracts the UMI and splits the read2 sequence at the exact linker-breakpoint (T-A) ligation junction. This step makes it possible to precisely map the breakpoint in the host genome. If the amplified fragment is short (less than approximately 200 nucleotides), the read1 sequence will read through the LTR-genomic junction to the added linker on the other end of the DNA molecule (200 nucleotides is within the Illumina sequence read length). In such cases, the scripts trim the linker sequence off of read1 for mapping, increasing the mapping accuracy.

After trimming both read1 and read2 sequences, the script also filters out genomic sequences that are less than 20 nucleotides long because it is difficult to map short host sequences precisely. We then merge the sequences. Because the sequence reads are derived from DNA fragments that are generated by PCR, there usually are many copies of each PCR fragment, all of which were generated from a single starting template. We merge identical reads to reduce the computational time used in mapping. Reads are merged if both the LTR-genome junction and the linker-breakpoint junction match exactly (based on the sequence of the 20 nucleotides on each side of the junction) and the fragments have the same UMI. The pipeline also filters the data based on the quality score of the sequence. Because the two ends of the trimmed DNA sequence, i.e., the LTR-genome junction and the linker-breakpoint junction, are the most important for precise mapping, we only accept data for which the sequencing average quality score for the first 20 nucleotides is greater than Q20 for both the trimmed read1 and read2 sequences.

The script creates fasta files of the unique trimmed high-quality read1-read2 pairs. The fasta files are then used for genome alignment. In addition to the host genomic sequences adjacent to the LTRs, we also expect to amplify the internal viral sequences that are adjacent to the LTRs. The internal junction sequences are expected to comprise about 50% of the reads. These sequences can serve as an important control for how well the amplification process works. Ordinarily, the internal viral sequences are excluded from the genome alignment step to speed up the alignment process. However, they can be used for a number of purposes, including studies of the sequence variation of the proviruses in the patient. The internal sequences can also be used as standards to estimate the number of infected cells and for the detection of aberrant integration products.

#### Genome alignment

The trimmed high-quality read1 and read2 sequences are then mapped to the genome using the command line BLAT alignment tool. Human genome hg19 is used in the example dataset because it permits backward compatibility with our earlier studies of integration site distributions. However, hg38 or other genome assemblies can used. There are different settings for BLAT that can be used to balance the speed and sensitivity of the mapping. Our standard settings are -ooc = 11.ooc -minScore = 16 -stepSize = 8, which give reasonable sensitivity without sacrificing speed. The program is efficient, and mapping can be done using a Mac OSX laptop. Alignments are done for each chromosome sequentially to reduce the demand for memory, which is not an issue if the mapping is done on a Unix server. A typical library sequenced on MiSeq with 20 million paired-end reads can be mapped to the human genome hg19 in a few hours on a MacBook Pro (Mid 2015 model with a 2.8 GHz Intel Core i7 and 16 GB memory). We also include other reference sequences during the alignment, for example the HIV genome. Including HIV sequences helps us identify the internal viral sequences that arise from priming on the other LTR, sequences that arise from autointegration, or other mechanisms that join the LTRs to internal HIV sequences. Other tools, such as Bowtie2, can also be used in the alignment step.

#### Post-alignment filtering and removal of artifacts

In the BLAT alignment results, each unique sequence is listed with all of the alignments to the reference sequences ranked by score. If the BLAT alignment is done chromosome by chromosome, the results are first combined to generate data for the whole genome and then each sequence was sorted by alignment score. We require the alignment to start within 3 bp from the trimmed read1 sequence (LTR-genomic junction) and to have a match of ≥95% identity for at least 20 nucleotides. Using a start within 3 bp tolerates minor sequence mismatches if there are differences in the DNA sequence of the patient and the reference genome. After this filtering step, some reads will have only a single alignment to the host genome. These are set aside for further analysis. Some reads still have multiple alignments to the genome. For these reads, if the difference between the best alignment score and the next best alignment score is greater than 10, the best alignment location is included for further analysis. If there are multiple alignments for a read whose alignment scores are similar, which can happen if a provirus is integrated in a repeat element, the read is considered “multimap” or “unmappable” to a unique location in the genome. There are also other reads that do not align to the genome, either because the reference genome is incomplete, or the reads are the result of some sort of artifact, or the sequence does not derive from the host genome. As described below, our bioinformatics pipeline takes another approach to find integration sites from clonally expanded cells even if the sites cannot be mapped to a unique sequence in the host genome. The same filtering criteria are used for read2 alignment at the linker-genomic junction.

After the alignments pass the filters described above, our pipeline compares the host genomic sequences at LTR-genomic DNA junctions and the linker-host breakpoint. If the read1 alignment and read2 host sequences are 1) on the same chromosome, 2) on the opposite strand, 3) within 1 kb of each other in the sequence of the host genome (the combined fragmenting and PCR steps do not produce amplified fragments longer than 1 kb), we conclude that the LTR/host junction likely represents a valid integration site. If the read1 alignment and read2 alignment are not on the same chromosome, or are too far apart, they were probably generated by ligation of fragments of host DNA, or by recombination during the PCR step. This is a common problem if restriction enzymes are used to fragment the genomic DNA before amplification, because the resulting DNA fragments have ligation compatible ends. The introduction of random DNA shearing followed by end-repair and T-dA ligation has almost completely eliminated the problems associated with the ligation of host DNA. However, recombination can occur during the PCR step. As noted above, and described in more detail in the Supplementary information, PCR recombination is a particular problem if there is a provirus integrated into a repetitive element, such as Alu [21], which provides a large number of potential recombination sites. This artifact is the primary reason that we do not use alignments based only on read1 sequences.

Reads that pass these initial checkpoints are subjected to additional filtering. Putative integration sites are checked for fuzz. When a single DNA template is amplified and sequenced thousands of times, PCR errors and/or sequencing errors will give rise to DNA sequences that are not an exact match for the original template. If the Illumina sequencing error rate is approximately 0.1% and the DNA is sequenced 10,000 times, there will be 10 reads that have a different base next to the LTR-junction or breakpoint-junction. Thus, there can appear to be different integration sites or breakpoints that are very close to each other even if the sequences are all derived from one bona fide integration site and breakpoint. In most cases, fuzz artifacts are easily identified because the true integration site and/or the breakpoint are present many more times than the derivative erroneous “nearby” sites or breakpoints, most (but not all) of which are within 1–3 bp. To help identify fuzz, we count and report the number of identical sequence reads in our final spreadsheets. Our bioinformatics pipeline looks for integration sites/breakpoints that are within 10 bp of one another for potential fuzz. The program takes into account the distance, the number of reads, and the UMI, to distinguish closely located sites/breakpoints that do, or do not, have an independent origin.

Integration sites and the corresponding breakpoints that have passed fuzz inspection are checked for mispriming. As is mentioned earlier and discussed at length in the Supplementary information, we have found that the viral primer can misprime on host DNA (which is present in vast excess, relative to proviruses, in patient samples), with as little as 9-nucleotide match at its 3′ end. Even though mispriming happens at a very low frequency, we sequence millions of PCR products, and 9-nucleotide matches are present by chance over 10,000 times in the genome. For that reason, we often see PCR products that are potentially caused by mispriming and our pipeline flags them for manual inspection.

#### Annotation and report generation

Once the integration sites and breakpoints have been filtered and the artifacts removed, the pipeline adds information to the report that helps with the interpretation of the data, including which LTR junction (3’LTR or 5’LTR) was used to identify the integration site, the orientation of the provirus (plus or minus with respect to the numbering of the genome), and if the provirus is read on the plus or minus strand of the reference chromosome. The integration sites are sorted by their breakpoints and UMIs and the read numbers are counted for each integration site. This step makes it easier to identify expanded clones. These data also help us identify genes that are preferred sites for integration and genes in which integrated proviruses can contribute to clonal expansion. Selection for proviruses integrated in (or near) a specific gene can be inferred by comparing the fraction of the proviruses that are integrated in the gene in a library from a patient, or patients, to the fraction of the proviruses integrated in the same gene in cultured cells analyzed shortly after they were infected.

#### Re-analyzing unmappable reads

Thus far, we have focused on integration sites that can be mapped to unique locations in the human genome. However, as was already discussed, some integration sites cannot be mapped to a unique site. One obvious example involves proviruses that are integrated in highly repetitive sequences. Our pipeline can be used to identify integration sites from clonally expanded cells even if they cannot be uniquely mapped. For this purpose, we use the UClust clustering program as a second screen for unmappable reads [26]. UClust takes the trimmed reads and sorts them from longest to shortest. The program sets the longest read as a seed, then compares all of the shorter reads to the seed. If significant identity is found for the next shorter read, it is included in the same cluster as the seed. If no significant match is found for the next shorter read, it becomes a new seed. The program continues to evaluate, in order, the shorter reads to all seeds. The program puts all valid reads into clusters regardless of whether they can be mapped to the host genome. We then sort the clusters by the number of breakpoints and map the consensus sequence of each cluster to the host genome using BLAT. If the consensus sequence can be mapped to the host genome and it has been previously identified, the cluster is confirmed as being mappable. We specifically look for clusters that cannot be mapped to unique locations. These clusters are then manually inspected. We used this approach to discover a large expanded clone, which was named “AMBI-1” for ambiguous-1, that carries an infectious provirus integrated into a sequence present more than once in the human genome. This provirus is responsible for the production of detectable viremia during suppressive ART [12, 22].

To demonstrate how the pipeline works, sequencing data from 3 patients were processed using the bioinformatics pipeline. Supplementary Table 1 shows the summary statistics of the analysis. Only a fraction of the total reads (13–27%) comprise uniquely mapped sequences that represent integration site junctions.

## Discussion

The earliest analyses of retroviral integration sites involved Southern blots [27–29], followed by the cloning and sequencing of individual integration sites [30–32]. These studies revealed the overall structure of the provirus and showed that there was little or no sequence specificity at the site of integration. However, a large-scale analysis of the distribution of proviruses in the host genome required the development of linker-mediated PCR [1, 8] which, together with the availability of the sequence of human (and other) genome(s), made it possible to determine the overall distribution of retroviral integration sites. Early studies showed that the global integration preferences for different types of retroviruses are different [33, 34]. For example, HIV prefers to integrate into the actively transcribed genes [1] and MLV prefers to integrate near transcription start sites and enhancers [7–9]. These differences in integration site preferences are due to the fact that the pre-integration complexes of the different types of retroviruses interact with different host factors, which have distinct distributions in the nucleus, and on chromatin [3, 4, 6, 35–38]. Initially, the sequences of the PCR products produced by the linker-mediated PCR reactions were determined using Sanger sequencing. The number of integration sites that were recovered increased dramatically with the advent of next generation sequencing. This increase in the sensitivity of integration site analysis made it possible to expand research from cells infected in culture to cells obtained from patients, in which the fraction of infected cells is often 1000-fold less than for cells infected in culture. Because the technology is powerful, there are an increasing number of studies that use integration site analysis, both in studies of cells infected in culture, and cells taken from patients, requiring the development of specialized public databases [39].

If an infected cell divides, an expanded clone of infected cells is produced, and all of the cells in the clone will have exactly the same integration site. Expanded clones can be identified by the presence, in a sample, of multiple DNA sequences with identical integration sites but different breakpoints. This approach has been used to show that a substantial fraction of the infected cells in patients are in large clones that derived from single infected cells. The first study which showed extensive clonal expansion of infected cells was done with samples from HTLV infected people [11]. Integration site analysis has also been used to show that clonal expansion of HIV infected cells is a major mechanism for the persistence of HIV infected cells in people on successful anti-retroviral therapy [12, 13, 40].

The combination of linker mediated PCR and next generation sequencing produce large datasets, and, unfortunately, these datasets always contain unwanted sequences that are not derived from real integration sites. If the sequencing data are not analyzed properly, artifactual integration sites can be generated which can lead to wrong conclusions. The most common artifacts include PCR recombination, cross-contamination, PCR mispriming, and fuzz.

PCR recombination can arise if there is incomplete copying of a DNA fragment during the elongation step. The partial DNA product can then act as a primer, leading to recombination if the partial DNA product base pairs to a site elsewhere in the genome. Subsequent amplification leads to the synthesis of a PCR recombinant. An example of this type of artifact, reported as a real integration site by Cohn et al. [21], is discussed in the Supplementary material (Figure S5). PCR recombination can be easily detected and filtered with a proper bioinformatics analysis pipeline. Identifying recombinant fragments relies on the fact that Illumina generates paired end sequences. In a correctly amplified fragment, the host sequences next to the LTR should be on the same chromosome, on the opposite strand, and be near the host sequences on the other end of the fragment. However, a recombinant product will often have ends that map to distant parts of the host genome.

Cross contamination is a common issue for procedures that involve high levels of PCR amplification and can be a significant issue for integration site analysis. Good general laboratory practices need to be followed. First, it is important to keep the starting material clean and free from contaminating DNA, particularly DNA that contains real, but extraneous, host/virus DNA junctions. We have developed standard procedures to catalog and identify patient samples, making sure that these samples, once cataloged, are kept in a low copy laboratory, one which is not used to store large amounts of HIV DNA, particularly from cultures of infected cells. It is particularly important to always keep patient samples, and the DNA derived from patient samples, in a laboratory that is well-separated from where either PCR amplification of host/virus DNA junctions or preparation of DNA from ex vivo infections takes place (high copy lab). For this reason, we use a low copy room to prepare host DNA for amplification and ensure that humans and/or contaminated lab coats are not allowed to move from any high copy lab space to the low copy lab space. Of the possible contaminants, the ones that are the most problematic (because they can be difficult to recognize in the bioinformatic analysis) are PCR products that were generated in the amplification of integration sites from other samples. Amplified samples can contain very large numbers of real, if unwanted, integration sites. These PCR products can be very efficiently amplified if any of the amplified material is introduced into a subsequent amplification reaction. Although it is not as obvious, there can also be problems with contamination of the viral primers and T-Linkers. Cross contamination of oligonucleotides can happen if several oligonucleotides are ordered at the same time. The indexes in the LTR primers/T-linkers must be rotated so that LTR primers/T-Linkers with unrelated indexes are used in sequential experiments. We have 48 different indexed LTR primers and T-Linkers on hand and run at least 2 experiments before reusing any index. As noted earlier, we do not use the same index for more than one sample in any run.

In addition to the contamination that can arise in the DNA preparation and amplification steps, there can be some carryover of amplified sequences between runs in an Illumina machine. In sequential runs on the same machine, this type of contamination can usually be detected if appropriate indexes were used; this is one of the reasons to switch indexes between samples. The pipeline settings allow for the use of multiple indexes. Each combination of indexes is searched and counted, allowing the user to check to see if any previously used indexes are present. We keep track of how many times we recover an amplified host/virus DNA junction sequence in all the Illumina sequencing runs, being particularly careful to check for amplified sequences that are present at low frequencies (once, or only a few times) to make sure that they were not carried over from a prior experiment.

Mispriming is a common problem in PCR. A primer can bind to sequences that are similar to the intended target sequences but are present elsewhere in the DNA in the sample. In integration site analysis, this can lead to incorrectly identifying mispriming sites as integration sites. Our reanalysis of the data in Cohn et al. [21] shows that as few as 9 bases of identity at the 3′ end of the viral-specific primer used in the first PCR step can cause mispriming. Frequently, mispriming sites are identified as integration sites in clonally expanded cells and are sometimes reported to be present at identical locations in samples from different patients [21]. Mispriming can be considerably reduced by careful primer design and by the use of nested PCR but cannot be completely eliminated. However, mispriming artifacts can be recognized through proper bioinformatic analysis. First, in the analytic pipeline, valid integration sites are those in which the sequence between the 3′ end of the nested LTR primer and the adjacent host sequence exactly matches the end of the HIV genome. Thus, the 3′ end of the nested viral primer should be far enough (at least 5 bp) from the end of the HIV provirus to allow a valid identification of the end of the HIV genome, making it possible to distinguish real integration sites from mispriming artifacts. Our pipeline flags any putative integration sites with nearby sequences matching >six of the ten 3′-most nucleotides of the LTR-specific primer for further inspection. Second, our analytic pipeline compares integration sites from multiple samples. Any exact matches to sites found in unrelated samples are likely to be derived from mispriming or cross-contamination.

There is also the problem we call fuzz. When a single piece of DNA is amplified and sequenced thousands of times, some errors will be introduced. These errors usually cause only minor problems in patient samples because the numbers of integration sites are relatively low, and sites within a few nucleotides of each other are rare, making it relatively easy to recognize integration site fuzz. More serious problems arise when breakpoints in the host genome are used to estimate the size of a clone based on the number of times a given integration site was recovered. In most cases, fuzz at a breakpoint arises when a highly amplified DNA fragment is sequenced many thousands of times, giving rise to a much smaller number of related sequences with slightly different breakpoints. If these secondary sequences are treated as real breakpoints it can lead to the misidentification of an integration site that was present only once in a sample as a clone. As was discussed earlier, keeping track of the number of times a fragment with a particular breakpoint has been sequenced, and the use of UMIs, makes it possible, in most cases, to determine if two closely related integration sites (or host breakpoints) derive from the same, or different, starting DNA templates.

There are some sequence reads that can be mapped to the genomes of species other than human. This could come from DNA contamination of the sample, or from sequencing-platform specific artifacts. For example, if unfiltered raw sequence reads are mapped to the GenBank all nucleotide collection using the Blast program, a large number of reads will map to the *Cyprinus carpio* (carp) genome. This happens if the Illumina sequencing adaptor sequences are not completely removed from the sequences. The carp genome assembly has a large number of Illumina adaptor sequences in it, and Blast maps the untrimmed Illumina adaptor sequences to the carp genome. We normally only map the sequence reads to the specific genome that was used in the study. Although most of our work involves HIV and the human genome, the experimental procedures and bioinformatics pipeline we describe here works for any retrovirus/host pair such as MLV in the human genome [7], HIV in the mouse genome [35, 41], or HIV or SIV in the macaque genome [42], with proper viral specific primers.

Even with all precautions we take, the confidence levels differ for different integration sites. If both the 3’LTR junction and 5’LTR junction of a provirus are identified, and they are 5 bp apart (the size of the target site duplication in the host genome that is created by the integration of HIV DNA), and in the opposite orientation relative to the host genome, we can be very confident that it is a real integration site. A highly expanded clone of infected cells will lead to the recovery of the same integration site many times, and the same integration site will be associated with multiple breakpoints in the appended host DNA. UMIs can be used to increase the confidence, when the breakpoints are close, that the PCR products originated from independent DNA templates. There is a potential problem if an integration site is only seen once, with a single breakpoint. If a DNA fragment that carries a particular integration site is rare (present only once or a few times in an Illumina dataset), it could be the result of contamination, and it is important not only to be very careful with the interpretation of the data, but, as has already been discussed, to design the workspace and amplification procedures to reduce contamination artifacts to a minimum. There are other published methods that give good advice for avoiding or reducing PCR/sequencing artifacts associated with the retroviral integration site analysis [43, 44].

Although some of the published protocols for retroviral integration site analysis use the LM- PCR approach, none of the available protocols was designed to accurately identify rare integration sites in samples from HIV infected individuals on therapy. For example, there are two published protocols for retroviral integration site analysis that were designed primarily for use with samples obtained from gene therapy patients [43, 45]. Although both protocols are similar ours, there are, in both cases, important differences. The clones that are of interest for gene therapy studies are normally both large and abundant. Neither of these protocols involves the isolation of both 5LTR/3LTR host/virus junctions. Unlike the proviruses of the lentiviral or MLV-based vectors that are used in the gene therapy, all which ordinarily have identical LTRs, the proviruses in samples from most HIV infected patients who are on long term therapy often have mutations in the LTRs. Our protocol increases the sensitivity of detection of integration sites from HIV infected patient samples by screening for both host/virus junctions, which is particularly important in samples where there are mutations in the LTRs. In addition, as we also have pointed out, the isolation of both host/virus junctions provides strong support that the integration site is real.

The protocol of Sherman et al. [43] includes a blocking LNA that is intended to prevent the amplification of sequences from the internal portions of the provirus. While that is probably helpful in a procedure that is intended to isolate large number of integration sites from gene therapy patients, as has already been discussed, allowing the amplification of the internal HIV sequences from patient samples can provide useful information. Importantly, in the kinds of samples we usually analyze, in which there are relatively few integration sites, the amplification of the internal fragments does not limit the recovery of the integration sites. There are other published methods that have been used for integration site analysis for HIV patient samples [13, 21]. Cohn et al. [21] described a protocol based on a modified translocation-capture sequencing method [46]. Even though, in principal, this method is similar to ours, because it is based on LM-PCR, and used similar bioinformatics tools such as BLAT and Bowtie for mapping, their implementation yielded significantly different results. As discussed in more detail in the supplementary material, we have re-analyzed their data and have identified several different types of artifacts that commonly arise when samples contain a small number of real integration sites are analyzed. We have designed the wet bench methods to minimize those problems and have also built into the analytical pipeline tools that will recognize, and can be used to remove, the residual errors that do arise.

In the analysis of our integration site data, we make considerable use of an integration site database https://rid.ncifcrf.gov/index.php that allows us to compare any new data we generate to all previously identified integration sites. This database can be used to help us check for artifacts and facilitates the comparison of the integration site data obtained in multiple studies.

## Conclusions

Combining linker-mediated PCR with next generation sequencing has revolutionized the analysis of retroviral integration sites and made it possible to ask and answer important questions about the behavior of infected cells in vivo. However, there are potential problems and pitfalls, particularly when the technology is used with samples in which the integration sites are rare, for example in samples from HIV infected people. We optimized the amplification protocol and bioinformatics pipeline, focusing on improving the sensitivity of amplifying and detecting bona fide integration sites. Our methods and bioinformatic analysis allow the user to distinguish valid integration sites from sequences that arise from the commonly encountered artifacts. Taking a combined approach makes it possible to obtain reliable integration site data, which can be used to answer many important questions, including, but not limited to, questions related to HIV persistence.

## Methods

An overview of the linker-mediated PCR, Illumina sequencing, and the bioinformatics pipeline is given in the Results. A complete step-by-step protocol describing the amplification and sequencing protocols is described here. Our experimental approach to identifying and quantifying HIV integration sites is based on linker-mediated amplification of sheared DNA from HTLV and HIV-infected cells [11, 12], modified to minimize artifacts due to mispriming, PCR recombination, cross-contamination, etc. General considerations are discussed in the main text.

### T-linker-UMI and primer design

Example sequences and the design of the primers and linkers used are shown in Figs. 1 and 3. The sequences of all oligonucleotides used are given in Fig. 2.

1. T-Linker-UMI
  The two strands are synthesized separately. The UMI portion of the linker is shown as Ns in Fig. 2. The UMI is synthesized using a mixture of equal amounts of dA, T, dC and dG at each position to create diverse sequences (Figs. 2 and 3).
2. First (outside) LTR primers
  The first PCR reaction is initiated using primers designed to match sequences near the ends of each LTR. Because the number of proviruses in patient samples is low, 1 μg of DNA is (usually) used in the amplification reactions, and the reactions are run in triplicate.
3. Nested PCR primers
  Nested PCR is used to increase the yield of LTR/host junction products relative to the large amounts of input genomic DNA. Nested PCR primers prime inside of (3′ of) the first PCR primers to increase both the yield and specificity of the amplification (Fig. 3). Illumina adaptors and the sequencing indexes are added during the nested PCR step. Nested primers for the LTR include an Illumina P5 grafting sequence, an 8 nucleotide i5 index, an Illumina PE1 SP sequence, an 8 nucleotide inline barcode, and 23–25 nucleotide LTR specific sequence (Fig. 2). Nested primers for the linker end are composed of an Illumina P7 grafting sequence, an 8 nucleotide i7 index and Illumina PE2 SP sequence (Fig. 2). The i5 and i7 indexes are used by the sequencer for initial sorting during fastq file generation and inline barcodes are used for secondary sorting during the de-barcoding step in our pipeline.

### DNA fragmentation, end repair, and linker ligation

We use the NEB Next Ultra II FS DNA Library Prep Kit for Illumina (New England Biolabs, Cat No. E7805S) for this step.

4) Anneal the short and long strands of the T-Linker-UMI
  This step is necessary if the linkers have undergone 3 freeze-thaw cycles.
  - Re-suspend the short and long linker strands in 1X low-EDTA TE pH 8.0 + 50 mM NaCl (STE buffer) at a concentration of 200 μM. Mix equal volumes of the short and long strands to give a final concentration of 100 μM each.
  - Incubate the linker mixture at 95 °C for 5 min.
  - Let the linker mixture cool down gradually for at least 30 min to room temperature then transfer to 4 °C for an additional 30 min. For long-term storage, make small aliquots and store at − 20 °C.
    ΔCRITICAL STEP: The efficiency of annealing the linkers can be compromised if the temperature drops too rapidly. Use a heat block or boiling water in a beaker to slow the cooling process. The quality of the library depends on the quality of linkers.
5) Sample DNA fragmentation and end repair.
  This step combines DNA fragmentation and end repair in one reaction. A one nucleotide 3′ dA overhang is added to the 3′ end of DNA fragment during this step. The 3′ dA overhang on the genomic DNA fragments and 3′ T overhang on the linker prevents genomic-genomic DNA and linker-linker ligation, increasing the efficiency of genomic-linker ligation.
  - Vortex the Ultra II FS enzyme mix and buffer for 5–8 s and place on ice prior to use.
  - Combine the following components in a sterile nuclease-free tube, vortex vigorously for 10 s, and place on ice.

**Table:** 

Component	Amount per reaction
DNA	1 μg
*Spike-in DNA control	1 ng
Ultra II FS enzyme mix	2 μl
5X Ultra II FS Reaction buffer	7 μl
Elution Buffer	up to 35 μl
total	35 μl

*Optional, DNA extracted from a cell clone grown culture carrying a single HIV provirus at a known position

ΔCRITICAL STEP: Make sure to vortex all components vigorously prior to incubation because the efficiency of DNA fragmentation can be compromised if the components are not mixed evenly and rapidly. This step needs to be finished quickly because the reaction will begin immediately and the timing of reaction determines the sizes of the fragments.

c) Samples are usually prepared in triplicate. The number of reactions (usually at least 3) run on each DNA sample depends on the number of proviruses in the sample, and the number of integration sites that are needed.
d) Place all samples in a PCR machine and run the program given below with the heated lid set to 75 °C. The length of the incubation at 37 °C determines the length of DNA fragments. Recommended incubation times can be found in the manufacturer’s instructions. We use 15 min at 37 °C, which is intended to produce fragments of ~ 500 bp.

**Table:** 

Cycle number	Temperature (°C)	Time (minutes)
1	37	15
2	65	30
3	4	∞

6) Ligate the DNA fragments and T-Linker-UMI
  - Combine the following components in a sterile nuclease-free tube, vortex and place the tube on ice.

**Table:** 

Component	Amount per reaction (μl)
fragmented DNA from last step	35
T-Linker-UMI (100 μM)	1.5
Ultra II ligation master mix	30
Ultra II ligation enhancer	1
total	67.5

b) Place all of the samples in a PCR machine and run the program given below with the heated lid set to ≥47 °C.

**Table:** 

Cycle number	Temperature (°C)	Time (minutes)
1	20	15
2	4	∞

7) Remove excess linkers and short DNA products
  This step is performed at room temperature using the Agencourt AMPure XP system (Beckman Coulter, Cat No. A63881).
  - Warm the AMPure beads at room temperature for at least 30 min prior to this step.
  - Vortex the AMPure XP beads extensively and vigorously to thoroughly resuspend the beads. Add 122 μl of the bead suspension (1.8x the volume of the ligation products) to the ligation products from previous step and mix well by pipetting at least 10 times.
  - Incubate for 5 min.
  - Separate the beads from the supernatant using a magnetic stand for 5 min or until the solution is clear.
  - Carefully remove the supernatant without disturbing the beads.
  - Wash the beads for 30 s with 300 μl of fresh 80% ethanol while the tubes are in the magnetic stand and carefully remove the supernatant.
  - Repeat step f).
  - Air dry the beads for 5 min with lid open or until liquid is not observed within the tubes while they are in the magnetic stand.
  - Add 70 μl of Qiagen Elution Buffer (EB) to the beads, mix by gentle pipetting to resuspend the beads.
  - Add 126 μl well-suspended AMPure XP beads and repeat step c) through h).
  - Elute the beads with 32 μl EB by extensively vortexing for 10 s and spinning for 5 s in a benchtop microcentrifuge.
  - Incubate for 5 min.
  - Separate the beads from the supernatant using a magnetic stand for 5 min or until the solution is clear.
  - Carefully transfer the supernatant into new tubes without disturbing the beads.
    ΔCRITICAL STEP: Bead contamination from this step could compromise the efficiency of the following PCR steps.
8) Use 2 μl of the DNA solution to determine the DNA concentration by Nanodrop.

ΔCRITICAL STEP: If the longer strand of the linker is not removed, it can act as a primer in the PCR reaction. When that happens, it will produce a second amplicon, with the same integration site, and the same breakpoint, but a different UMI. This problem explains why using only UMIs does not provide an accurate measure of whether two DNA fragments have an independent origin, and why using breakpoints and UMIs together gives the most accurate measure of clonal amplification.

### DNA library preparation

Cross contamination is a major concern for all next generation sequencing and is a particular problem in experiments in which the goal is to amplify something rare, like a proviral junction in a patient sample. With HIV integration sites, the sequence of the amplicon gives no information about which patient was the source. It is critically important to physically separate the lab into distinct areas in which low copy (pre-PCR) and high copy (post-PCR) samples are processed. The amplification and subsequent sequencing should be done in a high copy area with good practices that prevent unwanted contamination. As described in the text, is important to rotate the barcodes in a way that makes it possible to detect any cross-contamination that does occur.

9) First PCR
  This step selectively amplifies the host-virus integration junctions using one primer that matches the LTR sequence and a second primer that matches the single stranded portion of the linker (see Figs. 2 and 3). Each library will be split into 3 reactions.
  - Combine the following components in a sterile nuclease-free tube, mix and place the tube on ice. Both the 3’LTR and 5’LTR primers are used in the same reaction, thereby increasing the sensitivity of detection of the integration sites and, when both junctions are identified, providing strong evidence that the integration site is real.

**Table:** 

Component	Amount per reaction (μl)
DNA ligation products from last step	10
10X PCR buffer	5
MgCl_2_ (50 mM)	1.5
dNTPs (10 mM)	1
Platinum *Taq* DNA polymerase (ThermoFisher)	0.5
3LTR_Primer1 (10 μM)	2
5LTR_Primer1 (10 μM)	2
Linker_Primer1 (10 μM)	4
H_2_O	24
total	50

b) Place all samples in a PCR machine and run the program listed below with the heated lid set to 105 °C.

10) Cleanup of the products from the first PCR
  - Warm the AMPure beads at room temperature for at least 30 min prior to this step.
  - Vortex the AMPure XP beads extensively and vigorously to thoroughly resuspend the beads. Aliquot 90 μl bead suspension and mix well with PCR products (1.8x the volume of the PCR products) from previous step by pipetting at least 10 times.
  - Incubate for 5 min.
  - Separate the beads from the supernatant using a magnetic stand for 5 min or until the solution is clear.
  - Carefully remove the supernatant without disturbing the beads.
  - Wash the beads for 30 s with 300 μl of 70% ethanol while the tubes are in the magnetic stand, and carefully remove the supernatant.
  - Repeat f) two more times.
  - Air dry the beads for 5 min with the lid open or until liquid is not observed within the tubes while they are in the magnetic stand.
  - Elute the beads with 42 μl EB by extensively vortexing for 10 s and spin for 5 s using a benchtop centrifuge. Carefully transfer the supernatant into new tubes without disturbing the beads.
    ΔCRITICAL STEP: If beads from this step contaminate the supernatant, they will compromise the efficiency of the following PCR step.
11) Use 2 μl of the DNA solution to determine concentration by Nanodrop; pause and troubleshoot if the amount is too low. Low levels of PCR products can be a result of low quality of the original samples, primer mismatch, and/or inefficient recovery of the DNA from previous steps. Multiple primer sets with various lengths of matching sequences should be tested and purification steps should be optimized by adjusting the number of purification steps, washing steps, the amount of beads, and the amount of elution buffer.
12) Nested PCR
  This step uses an inner primer pair; one primer matches the sequence of the LTR and the other matches the sequence of the linker. The Illumina sequencing adapters and barcodes that identify the run are introduced in this step.
  - All of the samples are divided at this step to allow the 2 host junction fragments to be amplified separately using the nested primers specific for the 3′ and 5′ LTRs. Add the following components to two sterile nuclease-free tubes to amplify the 3′ and 5’LTR/host junctions, mix and place on ice.

**Table:** 

Component	Amount per reaction (μl)
first PCR products from last step	2
10X PCR buffer	2.5
MgCl_2_ (50 mM)	0.75
dNTPs (10 mM)	0.5
Platinum *Taq* DNA polymerase (ThermoFisher)	0.25
*3′/5’LTR_nested_Primer2 (10 μM)	2
Linker_nested_Primer2 (10 μM)	2
H_2_O	15
total	25

*Either the 3’LTR nested Primer2 or 5’LTR nested Primer2 will be added to the tube

b) Place all samples in a PCR machine and run the program given below with the heated lid set to 105 °C.

13) Exonuclease I treatment to degrade post-PCR primers
  Add 1.5 μl NEB Exonuclease I (M0293S) to each sample and mix well. Place all samples in a PCR machine and run the program given below with the heated lid set to 105 °C.

**Table:** 

Cycle number	Temperature (°C)	Time (minutes)
1	37	15
2	4	∞

14) Cleanup of the nested PCR products
  - Warm AMPure beads at room temperature for at least 30 min prior to this step.
  - Vortex AMPure XP beads extensively and vigorously to thoroughly resuspend the beads. Add 47.5 μl of bead suspension to the PCR products (1.8 times the volume) and mix well by pipetting at least 10 times.
  - Incubate for 5 min.
  - Separate the beads from the supernatant using a magnetic stand for 5 min or until the solution is clear.
  - Carefully remove the supernatant without disturbing the beads.
  - Wash the beads for 30 s with 300 μl 70% ethanol while the tubes are in the magnetic stand, and carefully remove the supernatant.
  - Repeat f) two more times.
  - Air dry the beads for 5 min with the lid open or until liquid is not observed within the tubes while they are in the magnetic stand.
  - Elute the beads with 25 μl EB by extensively vortexing for 10 s and spin for 5 s on a benchtop centrifuge. Carefully transfer the supernatant into new tubes without disturbing the beads.
    ΔCRITICAL STEP: If beads from this step contaminate the supernatant, they will compromise the efficiency of following qRT-PCR and sequencing steps.
15) Determine DNA concentrations by performing qRT-PCR using the KAPA Library Quantification Kit for Illumina Platforms (KAPA BIOSYSTEMS, KK4873) according to the manufacturer’s instructions. Be sure to use the version of the kit appropriate for your specific instrument. This step is used to determine how much DNA will be used for sequencing.

### DNA sequencing using the Illumina MiSeq system

Paired-end read DNA sequencing can be done on Illumina MiSeq, NextSeq, or HiSeq platforms. MiSeq gives high quality reads in quantities that generally are sufficient for most samples. NextSeq has higher throughput but the reads are of lower quality because it uses two color chemistry, and because integration site libraries are considered low complexity. HiSeq can generate higher throughput. Please note that the index setup is different for each platform even if the same index is used (see Illumina technical publications for the correct setup for each platform).

16) Thaw the MiSeq Reagent Kits v2 (300-cycles) (Illumina, cat. MS-102-2002) at 4 °C 24 h in advance. The reagents may also be thawed by placing the reagents cartridge in water (up to the max fill line) at room temperature for 1 h. Refrigerate the cartridge at 4 °C after thawing. Place the hybridization buffer (HT1) on ice. Reboot and wash the MiSeq system with 0.05% Tween20.
17) Sample denaturation and dilution to 20 pM
  - Combine the sample libraries from each tube proportionally according to their concentrations as determined by qRT-PCR and dilute to 2 nM using TE (0.05% Tween-20).
    ΔCRITICAL STEP: Be aware that the amount of the sample library used in each MiSeq run will affect the depth of sequencing and quality of the reads. If necessary, verify the concentration of the sample library by multiple methods and avoid loading too much of the sample on the MiSeq.
  - Combine 10 μl of the sample library and 10 μl 0.2 N NaOH in a sterile nuclease-free tube.
  - Vortex the mixture for 5 s and centrifuge at 280×g for 1 min.
  - Incubate the mixture at room temperature for 5 min.
  - Add 980 μl HT1 to the mixture and place it on ice.
18) PhiX library denaturation and dilution to 20 pM
  PhiX Control v3 (Illumina, cat. FC-110-3001) is used in this step. Once prepared, the PhiX library from this step can be stored at − 20 °C for 3 weeks.
  - Combine the following components in a sterile nuclease-free tube.

**Table:** 

Component	Amount per reaction (μl)
PhiX library (10 nM)	2
10 mM Tris-Cl (PH8.5, 0.1% Tween20)	3
0.2 N NaOH	5
total	10

b) Vortex the mixture for 5 s and centrifuge at 280×g for 1 min.
c) Incubate the mixture at room temperature for 5 min.
d) Add 990 μl HT1 to the mixture and place it on ice.

19) Combine sample library and PhiX library in a sterile nuclease-free tube as described below.

**Table:** 

Component	Amount per reaction (μl)
sample library (20 pM)	500
PhiX library (20 pM)	100
HT1	400
total	1000

20) Set up the MiSeq
  - Load 600 μl from the DNA library produced in the last step into the reagent cartridge.
  - Log in to the system and upload the sample sheet according to the manufacturer’s instructions.
    ΔCRITICAL STEP: While creating the sample sheet, be sure to input the i5 barcodes in LTR_Nested_Primer2 (Supplementary Table 2) as i5 indexes and input the reverse complement sequences of i7 barcodes in Linker_nested_Primer2 as i7 indexes. Demultiplexing of reads and generation of fastq files are based on the sample sheet. Failure to input accurate indexes in the sample sheet would result in placing what should be indexed reads into unindexed fastq files. (Caution: If setting up the NextSeq and HiSeq, the indexes are set up differently even though same primers are used).
  - Wash the sequencing chip with water, wipe with ethanol pads and lens paper until no trace of liquid is observed on the chip.
  - Load the chip, cartridge and buffer bottle into the MiSeq system according to the manufacturer’s instructions.
  - Start the run and expect it to finish in 24 h.

## Supplementary information

**Additional file 1: Table S1.** Summary of Bioinformatics Analysis of Example Datasets. **Table S2.** Primers Used for the Patient Samples. **Figure S1.** The most frequent integration site motif in the raw data of Cohn et al. The unfiltered raw dataset [1] comprising 80 million sites, was searched using MEME to find the most common short sequence motif, which is shown in sequence logo format aligned with the sequence of the LTR1, which is the first (outside) PCR primer used by [1] to amplify integration sites. **Figure S2.** Likely mispriming sites in the Cohn et al. integration dataset. Sequences (from hg19) that were within 50 bases of the 6719 integration sites determined by [1] (provided by the authors) were searched using BLAST for matches to their LTR1 primer. 1114 such sequences (± 30 bases of the reported integration site; arrow and dashed line) were found; 10 are shown aligned with LTR1 and with the matching bases highlighted in yellow. The chromosomal location of each site is shown to the left, with the number of patients reported to have a provirus at that site shown in parentheses. **Figure S3.** Plausible mechanisms for the erroneous identification of integration sites. The raw data used by [1] (NCBI accession number SRP045822) were searched for reads containing the cellular sequence 3′ of the reported integration site. No correct integration events were found, but several types of aberrant sequences were identified, including the 3 examples shown and labeled “raw read.” A. Double mispriming and PCR recombination. This sequence was most likely created by mispriming of LTR1 on the matching sequence (yellow) on chromosome 11 (magenta) as well as by LTR2 (green), and mispriming on a matching sequence on chromosome X (green), followed by recombination during PCR across the 8-base match indicated. B. Mispriming by a perfect fusion of sequences of LTR2-LTR1 on the same chromosome 11 sequence. C. Apparent correct integration (LTR2 followed by the 3′ 7 bases of the HIV-1 LTR1) two bases upstream of the reported integration site. The boxed sequence shows the 3′ most 7 nucleotides of the LTR, which are not in LTR2, but must be present in every correctly amplified integration site. **Figure S4.** The most common DNA sequence motif in the integration site datasets. Sequences ±50 base pairs of the reported integration sites from various studies are shown [1, 6, 11, 22], and 10,000 randomly chosen hg19 sequences and 10,000 Alu sequences were searched for common motifs using MEME [23]. The top hit in each case was aligned to the “integration site motif” of [1] (see text). The arrow above the sequence marks the site reported to be the preferred integration site [which is one nucleotide away from the site found at this site in the raw reads from patient 3, time point 3 (Figure S3). **Figure S5.** Sequence motifs adjacent to the integration site in various datasets. The patterns of preferred nucleotides in the host DNA immediately adjacent to the integration sites in patients reported by Maldarelli et al. [6] and Cohn et al. [1] were compared to data from cells infected in vitro (a PBMC dataset and a HeLa dataset). The sequence motifs were determined as previously described [8]. In the in vitro datasets, the target site nucleotides form a weak palindrome that matches what has been previously determined for HIV-1 using much smaller datasets [7, 8]. The preferred nucleotides in the Maldarelli dataset also match this motif. However, while the data of Cohn et al. shows some evidence of the palindrome; the sequence is weak and is not entirely symmetrical. The sequence from the Patient 3.3 sample (third time point) is obviously quite different from all the other data. **Figure S6**. Matching the Alu “integration sites” reported for the Patient 3.3 sample to the Alu consensus. The Alu consensus sequence is shown at the bottom. The quality of the match to the consensus is shown is the graph. The break in the match suggests the point in the sequence at which recombination frequently occurred.

## Data Availability

The Bioinformatics Software along with a small demo dataset is available at https://github.com/HughesLab-FNL/ISA-Analysis-Pipeline. The complete datasets used in this study is available through NCBI Sequence Read Archive (PRJNA597776: SRR10769116, SRR10769115, SRR10769114, SRR10769113, SRR10769112, SRR10769111 - which can all be found on the National Center for Biotechnology Information database).

## Abbreviations

ART: Anti-retroviral therapy
BLAT: BLAST-like alignment tool
ddPCR: Digital droplet PCR
HIV: Human immunodeficiency virus
LM-PCR: Linker-mediated PCR
MLV: Murine leukemia virus
UMI: Unique molecular identifier

## References

[1] Schroder AR, Shinn P, Chen H, Berry C, Ecker JR, Bushman F (2002). HIV-1 integration in the human genome favors active genes and local hotspots. Cell..

[2] Achuthan V, Perreira JM, Sowd GA, Puray-Chavez M, McDougall WM, Paulucci-Holthauzen A (2018). Capsid-CPSF6 interaction licenses nuclear HIV-1 trafficking to sites of viral DNA integration. Cell Host Microbe.

[3] Ciuffi A, Llano M, Poeschla E, Hoffmann C, Leipzig J, Shinn P (2005). A role for LEDGF/p75 in targeting HIV DNA integration. Nat Med.

[4] De Rijck J, de Kogel C, Demeulemeester J, Vets S, El Ashkar S, Malani N (2013). The BET family of proteins targets moloney murine leukemia virus integration near transcription start sites. Cell Rep.

[5] Gupta SS, Maetzig T, Maertens GN, Sharif A, Rothe M, Weidner-Glunde M (2013). Bromo- and extraterminal domain chromatin regulators serve as cofactors for murine leukemia virus integration. J Virol.

[6] Sharma A, Larue RC, Plumb MR, Malani N, Male F, Slaughter A (2013). BET proteins promote efficient murine leukemia virus integration at transcription start sites. Proc Natl Acad Sci U S A.

[7] De Ravin SS, Su L, Theobald N, Choi U, Macpherson JL, Poidinger M (2014). Enhancers are major targets for murine leukemia virus vector integration. J Virol.

[8] Wu X, Li Y, Crise B, Burgess SM (2003). Transcription start regions in the human genome are favored targets for MLV integration. Science..

[9] LaFave MC, Varshney GK, Gildea DE, Wolfsberg TG, Baxevanis AD, Burgess SM (2014). MLV integration site selection is driven by strong enhancers and active promoters. Nucleic Acids Res.

[10] Rosenberg N, Jolicoeur P, Coffin JM, Hughes SH, Varmus HE (1997). Retroviral pathogenesis. Retroviruses.

[11] Gillet NA, Malani N, Melamed A, Gormley N, Carter R, Bentley D (2011). The host genomic environment of the provirus determines the abundance of HTLV-1-infected T-cell clones. Blood..

[12] Maldarelli F, Wu X, Su L, Simonetti FR, Shao W, Hill S (2014). HIV latency. Specific HIV integration sites are linked to clonal expansion and persistence of infected cells. Science..

[13] Wagner TA, McLaughlin S, Garg K, Cheung CY, Larsen BB, Styrchak S (2014). HIV latency. Proliferation of cells with HIV integrated into cancer genes contributes to persistent infection. Science..

[14] De Ravin SS, Wu X, Moir S, Anaya-O'Brien S, Kwatemaa N, Littel P (2016). Lentiviral hematopoietic stem cell gene therapy for X-linked severe combined immunodeficiency. Sci Transl Med.

[15] Hacein-Bey-Abina S, Von Kalle C, Schmidt M, McCormack MP, Wulffraat N, Leboulch P (2003). LMO2-associated clonal T cell proliferation in two patients after gene therapy for SCID-X1. Science..

[16] Cavazzana-Calvo M, Payen E, Negre O, Wang G, Hehir K, Fusil F (2010). Transfusion independence and HMGA2 activation after gene therapy of human beta-thalassaemia. Nature..

[17] Aiuti A, Biasco L, Scaramuzza S, Ferrua F, Cicalese MP, Baricordi C (2013). Lentiviral hematopoietic stem cell gene therapy in patients with Wiskott-Aldrich syndrome. Science..

[18] Biffi A, Montini E, Lorioli L, Cesani M, Fumagalli F, Plati T (2013). Lentiviral hematopoietic stem cell gene therapy benefits metachromatic leukodystrophy. Science..

[19] Kustikova OS, Baum C, Fehse B (2008). Retroviral integration site analysis in hematopoietic stem cells. Methods Mol Biol.

[20] Bruner KM, Murray AJ, Pollack RA, Soliman MG, Laskey SB, Capoferri AA (2016). Defective proviruses rapidly accumulate during acute HIV-1 infection. Nat Med.

[21] Cohn LB, Silva IT, Oliveira TY, Rosales RA, Parrish EH, Learn GH (2015). HIV-1 integration landscape during latent and active infection. Cell..

[22] Simonetti FR, Sobolewski MD, Fyne E, Shao W, Spindler J, Hattori J (2016). Clonally expanded CD4+ T cells can produce infectious HIV-1 in vivo. Proc Natl Acad Sci U S A.

[23] Wiegand A, Spindler J, Hong FF, Shao W, Cyktor JC, Cillo AR (2017). Single-cell analysis of HIV-1 transcriptional activity reveals expression of proviruses in expanded clones during ART. Proc Natl Acad Sci U S A.

[24] Chun TW, Fauci AS (2012). HIV reservoirs: pathogenesis and obstacles to viral eradication and cure. AIDS..

[25] Finzi D, Blankson J, Siliciano JD, Margolick JB, Chadwick K, Pierson T (1999). Latent infection of CD4+ T cells provides a mechanism for lifelong persistence of HIV-1, even in patients on effective combination therapy. Nat Med.

[26] Edgar RC (2010). Search and clustering orders of magnitude faster than BLAST. Bioinformatics..

[27] Keshet E, Temin HM (1978). Sites of integration of reticuloendotheliosis virus DNA in chicken DNA. Proc Natl Acad Sci U S A.

[28] Hughes SH, Shank PR, Spector DH, Kung HJ, Bishop JM, Varmus HE (1978). Proviruses of avian sarcoma virus are terminally redundant, co-extensive with unintegrated linear DNA and integrated at many sites. Cell..

[29] Steffen D, Weinberg RA (1978). The integrated genome of murine leukemia virus. Cell..

[30] Vande Woude GF, Oskarsson M, Enquist LW, Nomura S, Sullivan M, Fischinger PJ (1979). Cloning of integrated Moloney sarcoma proviral DNA sequences in bacteriophage lambda. Proc Natl Acad Sci U S A.

[31] Shimotohno K, Temin HM (1980). No apparent nucleotide sequence specificity in cellular DNA juxtaposed to retrovirus proviruses. Proc Natl Acad Sci U S A.

[32] Hughes SH, Mutschler A, Bishop JM, Varmus HE (1981). A Rous sarcoma virus provirus is flanked by short direct repeats of a cellular DNA sequence present in only one copy prior to integration. Proc Natl Acad Sci U S A.

[33] Mitchell RS, Beitzel BF, Schroder AR, Shinn P, Chen H, Berry CC (2004). Retroviral DNA integration: ASLV, HIV, and MLV show distinct target site preferences. PLoS Biol.

[34] Derse D, Crise B, Li Y, Princler G, Lum N, Stewart C (2007). Human T-cell leukemia virus type 1 integration target sites in the human genome: comparison with those of other retroviruses. J Virol.

[35] Ferris AL, Wu X, Hughes CM, Stewart C, Smith SJ, Milne TA (2010). Lens epithelium-derived growth factor fusion proteins redirect HIV-1 DNA integration. Proc Natl Acad Sci U S A.

[36] Vandegraaff N, Devroe E, Turlure F, Silver PA, Engelman A (2006). Biochemical and genetic analyses of integrase-interacting proteins lens epithelium-derived growth factor (LEDGF)/p75 and hepatoma-derived growth factor related protein 2 (HRP2) in preintegration complex function and HIV-1 replication. Virology..

[37] Shun MC, Raghavendra NK, Vandegraaff N, Daigle JE, Hughes S, Kellam P (2007). LEDGF/p75 functions downstream from preintegration complex formation to effect gene-specific HIV-1 integration. Genes Dev.

[38] Schrijvers R, De Rijck J, Demeulemeester J, Adachi N, Vets S, Ronen K (2012). LEDGF/p75-independent HIV-1 replication demonstrates a role for HRP-2 and remains sensitive to inhibition by LEDGINs. PLoS Pathog.

[39] Shao W, Shan J, Kearney MF, Wu X, Maldarelli F, Mellors JW (2016). Retrovirus integration database (RID): a public database for retroviral insertion sites into host genomes. Retrovirology..

[40] Boritz EA, Darko S, Swaszek L, Wolf G, Wells D, Wu X (2016). Multiple origins of virus persistence during natural control of HIV infection. Cell..

[41] Wang H, Jurado KA, Wu X, Shun MC, Li X, Ferris AL (2012). HRP2 determines the efficiency and specificity of HIV-1 integration in LEDGF/p75 knockout cells but does not contribute to the antiviral activity of a potent LEDGF/p75-binding site integrase inhibitor. Nucleic Acids Res.

[42] Ferris AL, Wells DW, Guo S, Del Prete GQ, Swanstrom AE, Coffin JM (2019). Clonal expansion of SIV-infected cells in macaques on antiretroviral therapy is similar to that of HIV-infected cells in humans. PLoS Pathog.

[43] Sherman E, Nobles C, Berry CC, Six E, Wu Y, Dryga A (2017). INSPIIRED: a pipeline for quantitative analysis of sites of new DNA integration in cellular genomes. Mol Ther Methods Clin Dev.

[44] Berry CC, Nobles C, Six E, Wu Y, Malani N, Sherman E (2017). INSPIIRED: quantification and visualization tools for analyzing integration site distributions. Mol Ther Methods Clin Dev..

[45] Zhou S, Bonner MA, Wang YD, Rapp S, De Ravin SS, Malech HL (2015). Quantitative shearing linear amplification polymerase chain reaction: an improved method for quantifying lentiviral vector insertion sites in transplanted hematopoietic cell systems. Hum Gene Ther Methods.

[46] Klein IA, Resch W, Jankovic M, Oliveira T, Yamane A, Nakahashi H (2011). Translocation-capture sequencing reveals the extent and nature of chromosomal rearrangements in B lymphocytes. Cell..

