# Correction to: An analytical pipeline for identifying and mapping the integration sites of HIV and other retroviruses

**DOI:** 10.1186/s12864-020-06924-0

**Published:** 2020-07-29

**Authors:** Daria W. Wells, Shuang Guo, Wei Shao, Michael J. Bale, John M. Coffin, Stephen H. Hughes, Xiaolin Wu

**Affiliations:** 1 Cancer Research Technology Program, Leidos Biomedical Research, Inc., Frederick National Laboratory for Cancer Research, PO Box B, Frederick, MD 21702 USA; 2 Advanced Biomedical Computational Science, Leidos Biomedical Research, Inc., Frederick National Laboratory for Cancer Research, Frederick, MD USA; 3 HIV Dynamics and Replication Program, National Cancer Institute Frederick, National Institutes of Health, Frederick, MD USA; 4 Department of Molecular Biology and Microbiology, Tufts University, Boston, MA USA

**Correction to: BMC Genomics 21, 216 (2020)**

**https://doi.org/10.1186/s12864-020-6647-4**

Following publication of the original article [1], it was reported that Additional file 1 was missing the supplemental figures (Figs. S1-S6) referenced in the file. The complete Additional file including the missing figures is provided in this Correction.

## Supplementary information

**Additional file 1:.**
