# Supplementary material for: An analytical pipeline for identifying and mapping the integration sites of HIV and other retroviruses
Source: BMC Genomics. 2020 Mar 9;21:216. doi: 10.1186/s12864-020-6647-4 (PMC7063773; doi:10.1186/s12864-020-6647-4)
Supplement: Supplementary file 1 — Additional file 1: Table S1. Summary of Bioinformatics Analysis of Example Datasets. Table S2. Primers Used for the Patient Samples. Figure S1. The most frequent integration site motif in the raw data of Cohn et al. The unfiltered raw dataset [1] comprising 80 million sites, was searched using MEME to find the most common short sequence motif, which is shown in sequence logo format aligned with the sequence of the LTR1, which is the first (outside) PCR primer used by [1] to amplify integration sites. Figure S2. Likely mispriming sites in the Cohn et al. integration dataset. Sequences (from hg19) that were within 50 bases of the 6719 integration sites determined by [1] (provided by the authors) were searched using BLAST for matches to their LTR1 primer. 1114 such sequences (± 30 bases of the reported integration site; arrow and dashed line) were found; 10 are shown aligned with LTR1 and with the matching bases highlighted in yellow. The chromosomal location of each site is shown to the left, with the number of patients reported to have a provirus at that site shown in parentheses. Figure S3. Plausible mechanisms for the erroneous identification of integration sites. The raw data used by [1] (NCBI accession number SRP045822) were searched for reads containing the cellular sequence 3′ of the reported integration site. No correct integration events were found, but several types of aberrant sequences were identified, including the 3 examples shown and labeled “raw read.” A. Double mispriming and PCR recombination. This sequence was most likely created by mispriming of LTR1 on the matching sequence (yellow) on chromosome 11 (magenta) as well as by LTR2 (green), and mispriming on a matching sequence on chromosome X (green), followed by recombination during PCR across the 8-base match indicated. B. Mispriming by a perfect fusion of sequences of LTR2-LTR1 on the same chromosome 11 sequence. C. Apparent correct integration (LTR2 followed by the 3′ 7 bases of the HIV-1 LTR1) t [file 12864_2020_6647_MOESM1_ESM.docx]

***Supplementary Materials***

**An analytical pipeline for identifying and mapping retroviral DNA integration sites**

Daria W. Wells ^1^, Shuang Guo ^1^, Wei Shao ^2^, Michael J Bale^4^, John M. Coffin ^3^, Stephen H. Hughes ^4^, Xiaolin Wu ^1*^

^1^ Cancer Research Technology Program, Leidos Biomedical Research, Inc., Frederick National Laboratory for Cancer Research, Frederick MD

^2^ Advanced Biomedical Computational Science, Leidos Biomedical Research, Inc., Frederick National Laboratory for Cancer Research, Frederick, MD

^3^ Department of Molecular Biology and Microbiology, Tufts University, Boston MA, United States of America

^4^ HIV Dynamics and Replication Program, National Cancer Institute Frederick, National Institutes of Health, Frederick, MD

* Corresponding Author

**Artifacts that can affect interpretation of integration site data**

The very large amount of data that is produced by Illumina sequencing makes it impossible to attempt to analyze the data manually. It is possible to look at the results only after the raw reads have been processed by a computer. There is no good way to look directly at the Illumina data and ask whether the data are in agreement with what would have been expected, based on what is known about HIV integration. We will discuss the data from a published paper to illustrate the sorts of problems that can arise. These problems are discussed in a general way in the main text, what is given here are specific examples. This section is provided to help anyone who wants to use this type of approach understand how common problems can be recognized when the processed data are analyzed.

In the opening “Highlights” of the report by Cohn et al. (1), three primary conclusions are listed: 1) Sequencing integration sites identified clonally expanded and single HIV-1 integrations in human subjects; 2) Large families of clonally expanded HIV-1 infected cells are likely not part of the latent reservoir; 3) HIV-1 integrates near or into a 30 bp “INT-motif” found in Alu repeats. All of these principal conclusions are seriously flawed: 1) Sequencing of integration sites cannot distinguish single from amplified HIV-1 integrations in the small samples available from human subjects; 2) Many of the sites these authors identified as “clonally expended” were the result of mispriming artifacts rather than true integration events. We know that HIV-1 infected cells that have clonally expanded are part of the replication competent HIV-1 reservoir (2, 3) and 3) What was reported as multiple HIV-1 integrations into the 30 bp INT-motif in Alu repeats were the result of an artifact, most likely recombination during PCR.

**PCR Mispriming**

To investigate whether artifactual integration sites may have contaminated the dataset in Cohn et al. (1) and explain the unusually low level of integration in genes, we first inspected their raw sequence reads. We found a large number of reads that contained the sequence of LTR1, the viral primer used in first round PCR in their amplification strategy. Because the first round of PCR was followed by a second round of semi-nested PCR with a second viral specific primer, LTR2, which was internal to (3’ of) the LTR1 primer, LTR1 primer sequences should not have been present in any of the amplicons that were sequenced. The presence of a large number of sequences that matched the LTR1 primer in the amplified products (varying from a small fraction to as much as 90% in some samples) suggested that a substantial fraction of the products arose from mispriming.

Mispriming during PCR is a common problem, particularly when the target sequences constitute a very small fraction of the total DNA (in patient samples there is an average of about 1 provirus per 1,000 cells) (4). As discussed in the main text, additional bioinformatic processing must be used to identify and remove such artifacts. To investigate whether sequences that matched the LTR1 primer and may not have been derived from real integration sites and may have contributed to the list of 6719 integration sites reported by Cohn et al.(1), we extracted from the human genome (hg19), all of the sequences ±50 bases from each of the reported 6719 integration sites that were reported in the paper (provided by the authors). We performed a common sequence motif search using MEME sequence analysis software (5).This search revealed a very strong consensus motif, shown in Figure S1, which was a perfect match to the 3’ end of the LTR1 primer. We then expanded the search for matches to any part of the LTR1 primer. We found matches in the flanking sequences (±50 bases) for 1114 of the 6719 (16.9%) reported integration sites. All of the matches either spanned or were on the 3’ side of the reported integration site; in all cases the matches included the 9 3’ most bases of LTR1, as shown in the 10 examples in Figure S2. A search of the entire hg19 database revealed that the whole human genome contains 26,066 such matches, very close to the expected 21,634 [2.835*10^9^*2*(1/4^9^)], implying that it should be present (in either orientation) in about 0.1% of random 100-mers. Consistent with this expectation, only 106 (0.1%) such matches were found in the dataset of 100,000 random hg19 sites and only 4/1630 (0.2%) matches among the sites reported by Maldarelli et al. (6). The probability that the difference in the frequency at which this motif appears in the data reported by Cohn et al. (1) and the others is simply due to chance is extraordinarily small (P<2.21^-16^; Fisher’s exact or Binomial test).

Figure S3 shows three plausible mechanisms for generation of three of the sequences reported by Cohn et al (1). One example of a “clonally expanded integration site” likely due to mispriming is at (chr11: 41600857). One reason to doubt that this is a real integration site is that it was found in 5 samples from 4 different patients (P2.2, P2.3, P3.3, P7, P8). There was also another “clonally expanded integration site” that was reported to be 2bp away (chr11: 41600855) in two more samples (P2.5, P11.1). We retrieved all the raw sequence reads that mapped to this location and analyzed the alignment. Figure S3A shows a sequence that appears to be derived from at least 3 PCR artifacts: mispriming by LTR1 on a sequence in chromosome 11, mispriming by LTR2 on a sequence in the X chromosome, and PCR recombination that joined these two misprimed sequences. Figure S3B shows a sequence that appears to have been derived by the same LTR1 mispriming artifact on chromosome 11, followed (or perhaps preceded) by some sort of fusion event that directly joined the LTR2 and LTR1 primers (the generation of such “primer dimers” is a well-known, and common, PCR artifact). Figure S3C shows an apparently correct integration, but the site was misidentified (by 2 bases) in the final dataset.

These results imply that a significant fraction of the difference in the frequency of integration in genes reported by Cohn et al. (1), as compared to the results report by other researchers, derives from the failure of their analysis pipeline to remove common PCR artifacts from their dataset. Removing the sites with apparent mispriming at this sequence would increase the proportion of integration sites in genes from approximately 68% to about 73%, which is still lower than the fraction expected (ca. 80%) based on what has been reported in numerous studies from other laboratories.

**The “new” integration site sequence motif**

Cohn et al. (1) also reported what they claimed was a new motif that was a preferred site for HIV integration. They used MEME (5) to search hg19 sequences flanking the 6719 reported integration sites and found that 7% of the total sites were within 50 bases of the motif shown in Figure S4. This new sequence, which they refer to as the “INT-motif” is quite different from the previously reported consensus integration site sequence that flanks the HIV-1 integration site (7, 8). We repeated the MEME analysis by extracting genomic sequences +/-50 bp from the 6719 integration sites. In addition to the enrichment for the partial LTR1 primer motif discussed above, we confirmed the presence of the INT motif in 453 (7%) of the sites, of which 308 (5%) had an exact match to the inverse complement of the reported consensus sequence, GGGATTACAGG, following the 3’LTR, consistent with the results reported by Cohn et al. We then performed the same analysis on the sequences flanking the integration sites in the CD4+T-cell and patient libraries we generated for another study (not shown). We found that the same motif was present in a significant fraction (3-6%) of these 100 bp windows; however, in our libraries, the integration sites were found at different distances relative to the sequence motif, rather than being located at a distinct position. To understand the nature of this motif, we analyzed our random dataset. Multiple runs of 10,000 randomly selected sites showed that this sequence is the most common motif in the human genome, and that it is present within 50 bases of randomly chosen sites at a frequency of about 5%. As noted (1), the motif is found within the consensus *Alu* sequence, which we confirmed by showing that it was present in about 93% of 10,000 randomly selected *Alu* sequences, implying that it appears well over a million times in the human genome.

Further inspection showed that 286 of the *Alu*-related integration sites came from a single sample obtained from one of the patients at one specific time point (Pt3, time point 3), from which the authors report 737 “unique integration sites”. In approximately 90% of the reported *Alu*-related integration sites from the Pt3-3 sample, the provirus was integrated into the 20^th^ position of the motif (Figure S4). However, all of these proviruses were integrated in the same orientation. In all the other patient samples, the orientation of the integration sites relative to the *Alu* motif was random, and the frequency at which the integration sites appeared, relative to the *Alu* motif, and to the “preferred site,” also appeared to be random. To understand the discrepancy between the orientation data for this single patient and the rest of the dataset, we also searched libraries made from infected CD4+ T-cells, HeLa cells, and other patient datasets for integration sites in the same orientation and at the same site in the *Alu* motif. We did find a few such integrations in all these datasets, in all cases at a frequency very near what would be expected if the integration sites were randomly distributed within this region. Importantly, if the Alu-related sites from this single Pt3-3 sample are excluded from the Cohn et al. dataset (1), then the frequency at which the Alu-related sequence motif is associated with HIV integration sites in their dataset falls below the random control.

Additionally, Cohn et al. (1) apparently misinterpreted the previous publications that described a weakly palindromic set of nucleotides that flank the HIV integration site (7, 8). This motif flanks the integration sites in the CD4+T-cell, HeLa, and Maldarelli et al. patient datasets, but could not be found in the Cohn et al. dataset (Figure S5). The fact that this motif is symmetric, taken together with insights from the crystal structure of the intasome of another retrovirus, prototype foamy virus (9), strongly supports the idea that integration of retroviral DNA, including HIV DNA integration, is not directional or specific for one LTR or the other. However, the asymmetric consensus integration site described in Cohn et al. (1), would require that HIV integration be directional and LTR specific.

All of these observations: the presence of a “preferred” integration site in only one of the samples from the patients; the very high frequency of the motif in the human genome; the absence of any evidence of clonal amplification of cells that have proviruses inserted at this site; the asymmetry of the site; and the discordance with previous reports, strongly suggest that there are other mechanisms that explain these findings. The most likely explanation is that, during PCR, there was extensive recombination within the Alu sequence that caused a particular integration site within one Alu sequence to become associated with DNA from some of the many other Alu sequences in various chromosomal locations. This interpretation is consistent with analysis of 200 integration events at this site, obtained from the raw data from Patient 3-3, which implies that there was a major site of recombination 24 nucleotides 3’ of the end of the LTR (Figure S6). The frequency of this artifact may well have been enhanced by the fact that Cohn et al. used both a linear amplification step and a BglII digestion step (to avoid amplifying internal viral sequences) in their protocol. Whatever the specific mechanism, the net result is that the overall distribution of the described events in which proviruses appeared to have been inserted into a particular position in a number of different *Alu* sequences was due to single integration event followed by recombination during PCR. Thus, the claimed distribution of integration sites reflects the distribution of the *Alu* elements in the genome and the inclusion of these artifactual integration sites in the dataset almost certainly also contributed to obtaining an incorrect frequency for HIV‑1 integration in genes.

**Other Problems with the Cohn et al. data.**

In the course of reanalyzing the data of Cohn et al. (1), we also uncovered a number of other issues. First, the bioinformatics pipeline used for processing sequences obtained by Cohn et al. (1) did not identify a specific integration site sequence. Rather, according to their Experimental Procedures, “adjacent (within 50 nucleotides) integration sites were merged,” making it impossible to identify an exact host: HIV junction sequence. Retroviral DNA integration is nearly always precise, joining the 3’-terminal CA of the LTR to a target site in cell DNA, with no extraneous bases (10). We and others have identified numerous junction sequences of integration sites in HIV infected cells (6, 11) and in cells infected with other retroviruses (12-16). Our analysis pipeline discards the few sequences that do not meet the exact integration criterion. Examination of the raw data of Cohn et al. reveals very large numbers of sequences with small numbers of nucleotides of unknown origin between a correct LTR and identifiable host cell DNA sequence. The analytical pipeline used by Cohn et al. (an outline of which was provided by the authors), allowed such sequences to be passed through as long as the unidentified sequence was not present in the HIV genome. As stated in the paper, the protocol and analytic pipeline used were derived from ones developed for another purpose—mapping chromosomal translocation break points (17), where the presence of unidentifiable bases at the junction site is apparently acceptable. However, in evaluating HIV integration sites, a failure to remove such sequences appeared to allow misidentification of artifacts as HIV DNA integration sites.

Second, further analysis of the quantitative aspects of the Cohn et al. data suggested that there was an issue with the recovery of integration sites. Cohn et al. reported isolating almost 2x10^4^ integration sites starting with 10^6^ PBMC from the 5^th^ time point sample from patient 2 (their Table S3), accounting for more than 1/3 of the total independent integration sites. In patient samples, most infected cells carry a single provirus (18). If the recovery rate of the integration sites in the sample is approximately 10%, which is, based on our experience, a good recovery, then the actual number of infected cells in the sample analyzed by Cohn et al. would have been approximately 2x10^5^ out of the 10^6^ cells in the starting sample. This is highly improbable, because patients on long-term therapy generally have less than 10^3^ infected cells per 10^6^ PBMC (4). Even if their recovery of sites in the sample was 100%, these data indicate the recovery of 20-fold more integration sites than the expected number of proviruses in the sample.

There are other issues surrounding identification and quantitation of the HIV integration sites by Cohn et al. (1) that require re-assessment. Of particular concern is the claim that clonally amplified cells do not contain intact proviruses and are not part of the reservoir. The authors distinguished “singles” from clonal expanded cells and concluded that clonally expanded cells are highly unlikely to have intact proviruses. However, it is not possible to accurately identify any integration sites as “singles” in samples taken from a patient because only a very small fraction of the billion or so infected cells that are present in the whole body is sampled. Moreover, in integration site analysis, only a fraction of the integration sites is recovered from these relatively small samples. Even extensively amplified sites may be present only once in integration site libraries prepared from patient samples.

Cohn et al. (1) concluded that it is unlikely that there are replication competent proviruses in clonally expanded cells based on results obtained in experiments intended to amplify 5’ viral sequences from proviruses detected using 3’ LTR primers. They reported an analysis of 75 expanded clones and reported that no full-length HIV proviruses were detected. However, in nearly half of the 75 examples, the 5’ end of the viral genome could not be amplified at all. These 5’ amplification reactions could have failed for a number of reasons, including a primer mismatch in the viral sequences, or, more likely, the possibility that a significant fraction of these 75 integration sites were the result of mispriming events for which there was no real integration site at the reported position. A large fraction of the sites they attempted to verify were found in samples from multiple patients, a strong indication that these “sites” arose as mispriming events.

Even if all 75 proviruses actually were present, and all were defective, a failure to find an intact provirus in the 75 amplified clones would not be unexpected. It has been estimated that as much as 98% of the proviruses present in patients on long-term ART are defective (19, 20). Therefore, the probability of getting a negative result by chance from looking at 75 proviruses is 0.22. If a more accurate count of the number of defective proviruses that were actually present in amplified cells in the patient sample was 30 (the number that could be amplified), then the probability, if 93% are defective, of finding all are defective by chance is a statistically insignificant 0.11. Thus, the analyses by Cohn et al. cannot be used to conclude that none of the proviruses present in clonally-expanded cells is intact and replication competent. Furthermore, in patient 1 of Maldarelli et al. (6), one of the most highly amplified proviruses, found in 3-5% of infected cells, encodes a completely intact provirus. Cells that carry this provirus produce infectious virus following ex vivo activation, and the provirus responsible for the majority of plasma virus found in the patient even after more than 10 years on therapy (21). As this counterexample shows, clonally expanded cells can and do give rise to infectious HIV.

**References**

1. Cohn LB, Silva IT, Oliveira TY, Rosales RA, Parrish EH, Learn GH, et al. HIV-1 integration landscape during latent and active infection. Cell. 2015;160(3):420-32.

2. Simonetti FR, Sobolewski MD, Fyne E, Shao W, Spindler J, Hattori J, et al. Clonally expanded CD4+ T cells can produce infectious HIV-1 in vivo. Proc Natl Acad Sci U S A. 2016;113(7):1883-8.

3. Einkauf KB, Lee GQ, Gao C, Sharaf R, Sun X, Hua S, et al. Intact HIV-1 proviruses accumulate at distinct chromosomal positions during prolonged antiretroviral therapy. J Clin Invest. 2019.

4. Besson GJ, Lalama CM, Bosch RJ, Gandhi RT, Bedison MA, Aga E, et al. HIV-1 DNA decay dynamics in blood during more than a decade of suppressive antiretroviral therapy. Clin Infect Dis. 2014;59(9):1312-21.

5. Bailey TL, Elkan C. Fitting a mixture model by expectation maximization to discover motifs in biopolymers. Proc Int Conf Intell Syst Mol Biol. 1994;2:28-36.

6. Maldarelli F, Wu X, Su L, Simonetti FR, Shao W, Hill S, et al. HIV latency. Specific HIV integration sites are linked to clonal expansion and persistence of infected cells. Science. 2014;345(6193):179-83.

7. Holman AG, Coffin JM. Symmetrical base preferences surrounding HIV-1, avian sarcoma/leukosis virus, and murine leukemia virus integration sites. Proc Natl Acad Sci U S A. 2005;102(17):6103-7.

8. Wu X, Burgess SM. Integration target site selection for retroviruses and transposable elements. Cell Mol Life Sci. 2004;61(19-20):2588-96.

9. Maertens GN, Hare S, Cherepanov P. The mechanism of retroviral integration from X-ray structures of its key intermediates. Nature. 2010;468(7321):326-9.

10. Craigie R, Bushman FD. HIV DNA integration. Cold Spring Harbor perspectives in medicine. 2012;2(7):a006890.

11. Wagner TA, McLaughlin S, Garg K, Cheung CY, Larsen BB, Styrchak S, et al. HIV latency. Proliferation of cells with HIV integrated into cancer genes contributes to persistent infection. Science. 2014;345(6196):570-3.

12. Cook LB, Rowan AG, Melamed A, Taylor GP, Bangham CR. HTLV-1-infected T cells contain a single integrated provirus in natural infection. Blood. 2012;120(17):3488-90.

13. Gillet NA, Malani N, Melamed A, Gormley N, Carter R, Bentley D, et al. The host genomic environment of the provirus determines the abundance of HTLV-1-infected T-cell clones. Blood. 2011;117(11):3113-22.

14. Justice Jt, Malhotra S, Ruano M, Li Y, Zavala G, Lee N, et al. The MET gene is a common integration target in avian leukosis virus subgroup J-induced chicken hemangiomas. Journal of virology. 2015;89(9):4712-9.

15. Kim HH, van den Heuvel AP, Schmidt JW, Ross SR. Novel common integration sites targeted by mouse mammary tumor virus insertion in mammary tumors have oncogenic activity. PloS one. 2011;6(11):e27425.

16. Melamed A, Witkover AD, Laydon DJ, Brown R, Ladell K, Miners K, et al. Clonality of HTLV-2 in natural infection. PLoS pathogens. 2014;10(3):e1004006.

17. Klein IA, Resch W, Jankovic M, Oliveira T, Yamane A, Nakahashi H, et al. Translocation-capture sequencing reveals the extent and nature of chromosomal rearrangements in B lymphocytes. Cell. 2011;147(1):95-106.

18. Josefsson L, Palmer S, Faria NR, Lemey P, Casazza J, Ambrozak D, et al. Single cell analysis of lymph node tissue from HIV-1 infected patients reveals that the majority of CD4+ T-cells contain one HIV-1 DNA molecule. PLoS Pathog. 2013;9(6):e1003432.

19. Bruner KM, Wang Z, Simonetti FR, Bender AM, Kwon KJ, Sengupta S, et al. A quantitative approach for measuring the reservoir of latent HIV-1 proviruses. Nature. 2019;566(7742):120-5.

20. Ho YC, Shan L, Hosmane NN, Wang J, Laskey SB, Rosenbloom DI, et al. Replication-competent noninduced proviruses in the latent reservoir increase barrier to HIV-1 cure. Cell. 2013;155(3):540-51.

21. Simonetti FR, Sobolewski MD, Fyne E, Shao W, Spindler J, Hattori J, et al. Clonally Expanded CD4+ T-Cells Can Produce Infectious HIV-1 in vivo. Proc Natl Acad Sci U S A. 2016;In Press.

22. Sherrill-Mix S, Lewinski MK, Famiglietti M, Bosque A, Malani N, Ocwieja KE, et al. HIV latency and integration site placement in five cell-based models. Retrovirology. 2013;10:90.

23. Bailey JR, Sedaghat AR, Kieffer T, Brennan T, Lee PK, Wind-Rotolo M, et al. Residual human immunodeficiency virus type 1 viremia in some patients on antiretroviral therapy is dominated by a small number of invariant clones rarely found in circulating CD4+ T cells. Journal of virology. 2006;80(13):6441-57.

Supplementary Table 1. Summary of Bioinformatics Analysis of Example Datasets

|  | PT1_3LTR | PT1_5LTR | PT2_3LTR | PT2_5LTR | PT3_3LTR | PT3_5LTR |
| --- | --- | --- | --- | --- | --- | --- |
| Read 1 file size | 350.7 MB | 1.18 GB | 942.9 MB | 636.6 MB | 562.9 MB | 459.3 MB |
| Read 2 file size | 388.6 MB | 1.34 GB | 1.06 GB | 718.12 MB | 653.4 MB | 542.7 MB |
| Total paired-end reads | 4,155,600 | 13,358,612 | 11,495,483 | 7,265,423 | 7,831,881 | 6,065,234 |
| Mapped internal reads | 654,915 | 3,061,803 | 3,047,849 | 2,349,219 | 1,924,470 | 2,107,379 |
| Uniquely mapped reads (non-internal) | 1,123,100 | 3,494,235 | 1,460,654 | 1,004,720 | 1,353,571 | 1,069,580 |
| Percent of Uniquely mapped reads to hg19 | 27% | 26% | 13% | 14% | 17% | 18% |
| Multi- mapped reads (1 or both of pair) | 173,929 | 22,801 | 14,074 | 11,136 | 5,795 | 4,538 |
| OS10 Laptop 1_demultiplex.pl real run time | 2m59.052s | 10m11.824s | 5m53.312s | 3m57.828s | 4m42.743s | 4m16.231s |
| OS10 Laptop 2_blat_pipeline.pl real run time | 13m42.258s | 55m26.368s | 31m59.685s | 30m29.182s | 15m45.800s | 20m1.537s |
| OS10 Desktop 1_demultiplex.pl real run time | 3m11.700s | 10m49.712s | 6m29.733s | 4m39.544s | 5m14.305s | 4m38.947s |
| OS10 Desktop 2_blat_pipeline.pl real run time | 13m38.970s | 58m29.034s | 33m47.956s | 31m3.381s | 16m47.223s | 21m11.047s |
| Linux Cluster 1_demultiplex.pl real run time | 3m6.622s | 10m48.534s | 6m13.454s | 4m27.500s | 5m15.736s | 4m33.954s |
| Linux Cluster 2_blat_pipeline.pl real run time | 12m6.362s | 49m0.430s | 28m42.799s | 27m49.851s | 14m20.487s | 18m29.554s |
| Footnotes  All execution times were done using single core by default with Perl and BLAT even if multiple cores were available in the system.  OSX Laptop settings: MacBookPro laptop, 2.8 Ghz Intel Quad Core i7, 16 GB memory, SSD drive, MacOS10.14  OSX Desktop settings: MacPro desktop, 3.7 Ghz Intel Quad-core Xeon E5, 32 Gb memory, SSD drive, MacOS10.14  Unix Server settings: Linux cluster, Intel Xenon 2.7Ghz,total 3276 CPU cores. | | | | | | |

**
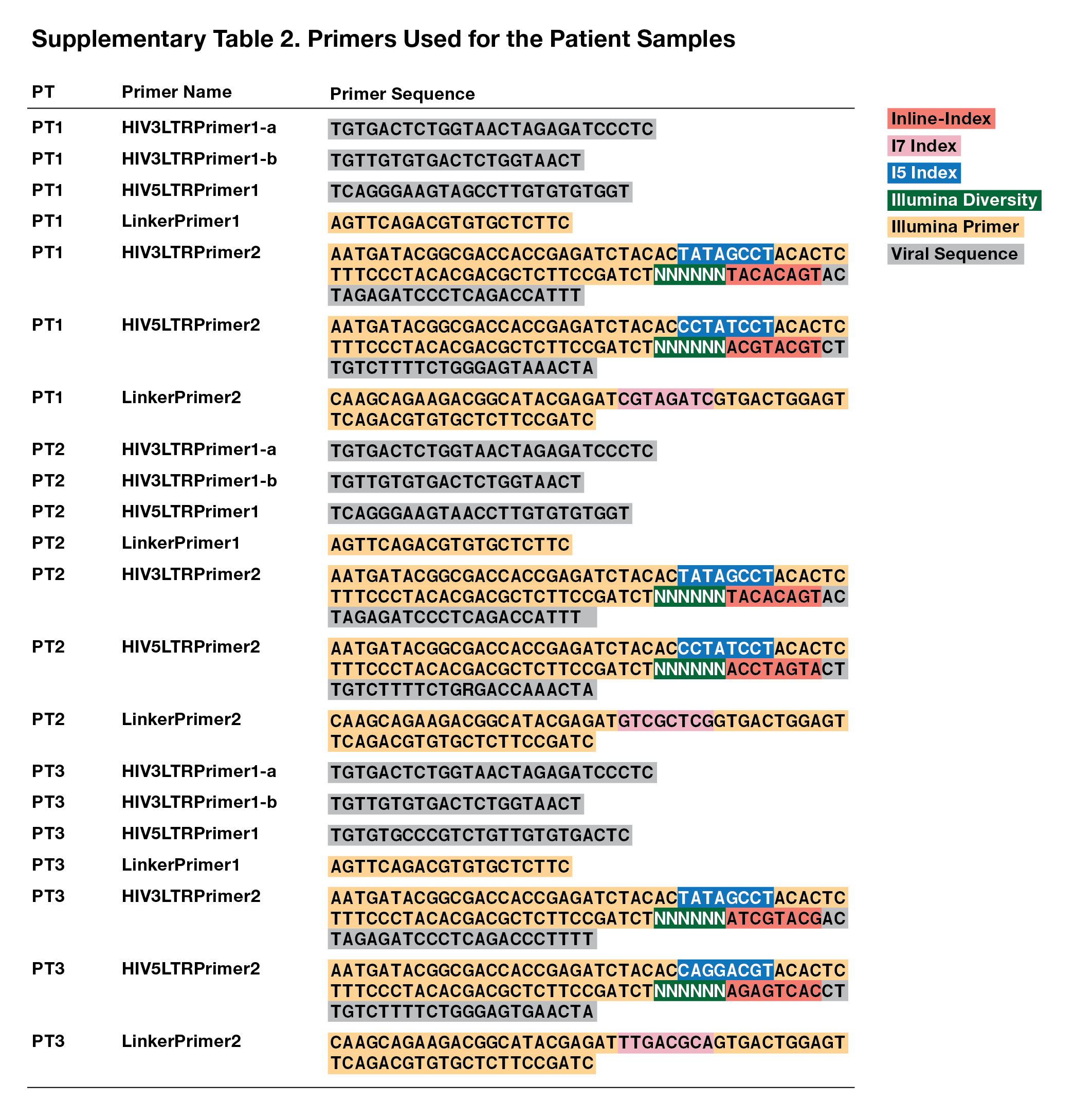
**

**Figure Legends**

**Figure S1.** The most frequent integration site motif in the raw data of Cohn et al. The unfiltered raw dataset (1) comprising 80 million sites, was searched using MEME to find the most common short sequence motif, which is shown in sequence logo format aligned with the sequence of the LTR1, which is the first (outside) PCR primer used by (1) to amplify integration sites.

**Figure S2**. Likely mispriming sites in the Cohn et al. integration dataset. Sequences (from hg19) that were within 50 bases of the 6719 integration sites determined by (1) (provided by the authors) were searched using BLAST for matches to their LTR1 primer. 1114 such sequences (± 30 bases of the reported integration site; arrow and dashed line) were found; 10 are shown aligned with LTR1 and with the matching bases highlighted in yellow. The chromosomal location of each site is shown to the left, with the number of patients reported to have a provirus at that site shown in parentheses.

**Figure S3.** Plausible mechanisms for the erroneous identification of integration sites. The raw data used by (1) (NCBI accession number SRP045822) were searched for reads containing the cellular sequence 3’ of the reported integration site. No correct integration events were found, but several types of aberrant sequences were identified, including the 3 examples shown and labeled “raw read.” A. Double mispriming and PCR recombination. This sequence was most likely created by mispriming of LTR1 on the matching sequence (yellow) on chromosome 11 (magenta) as well as by LTR2 (green), and mispriming on a matching sequence on chromosome X (green), followed by recombination during PCR across the 8-base match indicated. B. Mispriming by a perfect fusion of sequences of LTR2-LTR1 on the same chromosome 11 sequence. C. Apparent correct integration (LTR2 followed by the 3’ 7 bases of the HIV-1 LTR1) two bases upstream of the reported integration site. The boxed sequence shows the 3’ most 7 nucleotides of the LTR, which are not in LTR2, but must be present in every correctly amplified integration site.

**Figure S4.** The most common DNA sequence motif in the integration site datasets. Sequences ±50 base pairs of the reported integration sites from various studies are shown (1, 6, 11, 22), and 10,000 randomly chosen hg19 sequences and 10,000 Alu sequences were searched for common motifs using MEME (23). The top hit in each case was aligned to the “integration site motif” of (1) (see text). The arrow above the sequence marks the site reported to be the preferred integration site [which is one nucleotide away from the site found at this site in the raw reads from patient 3, time point 3 (Figure S3).

**Figure S5.** Sequence motifs adjacent to the integration site in various datasets. The patterns of preferred nucleotides in the host DNA immediately adjacent to the integration sites in patients reported by Maldarelli et al. (6) and Cohn et al. (1) were compared to data from cells infected in vitro (a PBMC dataset and a HeLa dataset). The sequence motifs were determined as previously described (8). In the in vitro datasets, the target site nucleotides form a weak palindrome that matches what has been previously determined for HIV-1 using much smaller datasets (7, 8). The preferred nucleotides in the Maldarelli dataset also match this motif. However, while the data of Cohn et al. shows some evidence of the palindrome; the sequence is weak and is not entirely symmetrical. The sequence from the Patient 3.3 sample (third time point) is obviously quite different from all the other data.

**Figure S6**. Matching the Alu “integration sites” reported for the Patient 3.3 sample to the Alu consensus. The Alu consensus sequence is shown at the bottom. The quality of the match to the consensus is shown is the graph. The break in the match suggests the point in the sequence at which recombination frequently occurred.
