# Supplementary material for: Correction to: An analytical pipeline for identifying and mapping the integration sites of HIV and other retroviruses
Source: BMC Genomics. 2020 Jul 29;21:517. doi: 10.1186/s12864-020-06924-0 (PMC7388206; doi:10.1186/s12864-020-06924-0)
Supplement: Supplementary file 1 — Additional file 1:. [file 12864_2020_6924_MOESM1_ESM.zip › WuBMCGenomics_Figs_supp.pptx]

## Slide 1
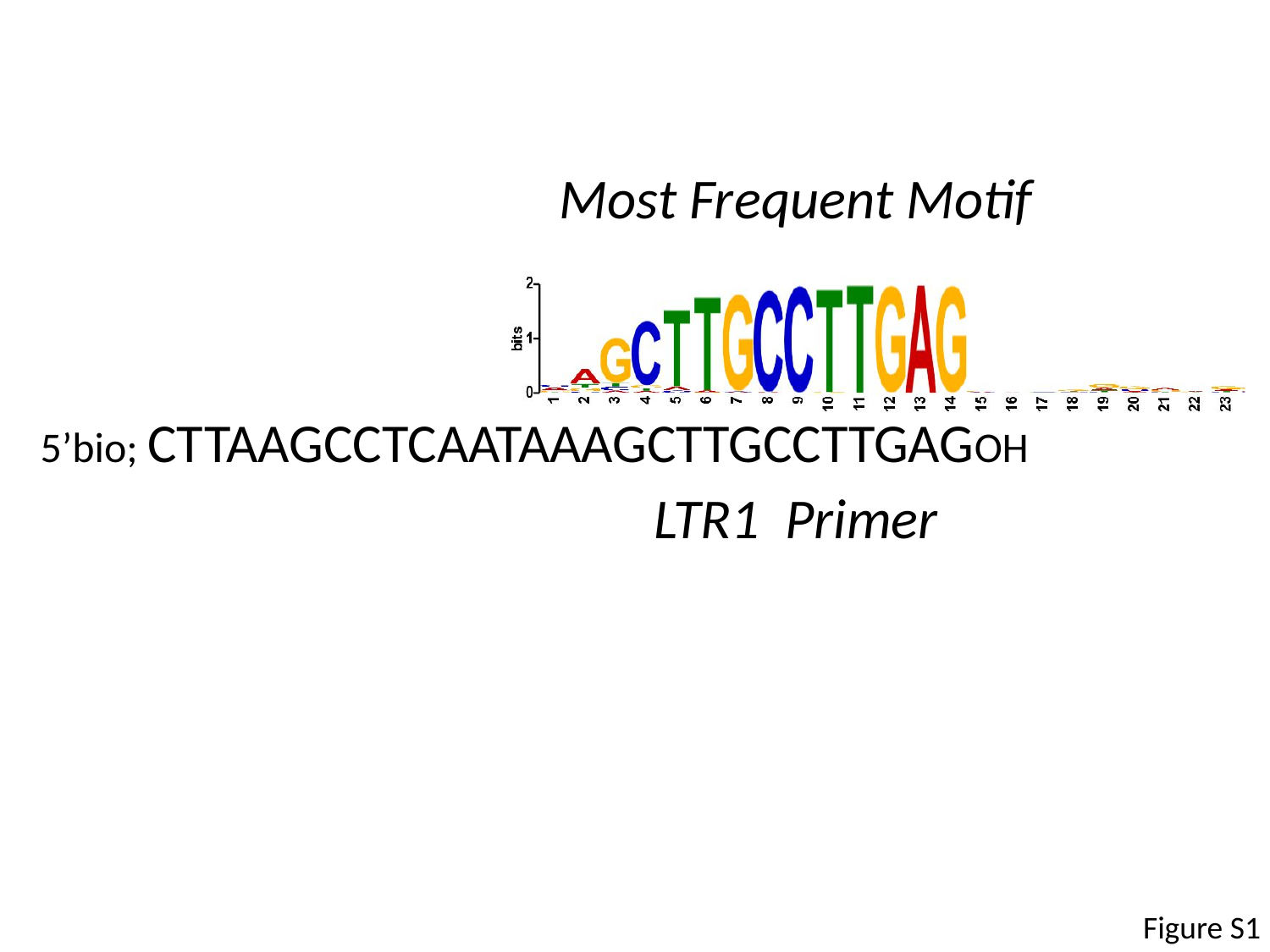

Most Frequent Motif
5’bio; CTTAAGCCTCAATAAAGCTTGCCTTGAGOH
LTR1 Primer
Figure S1

## Slide 2
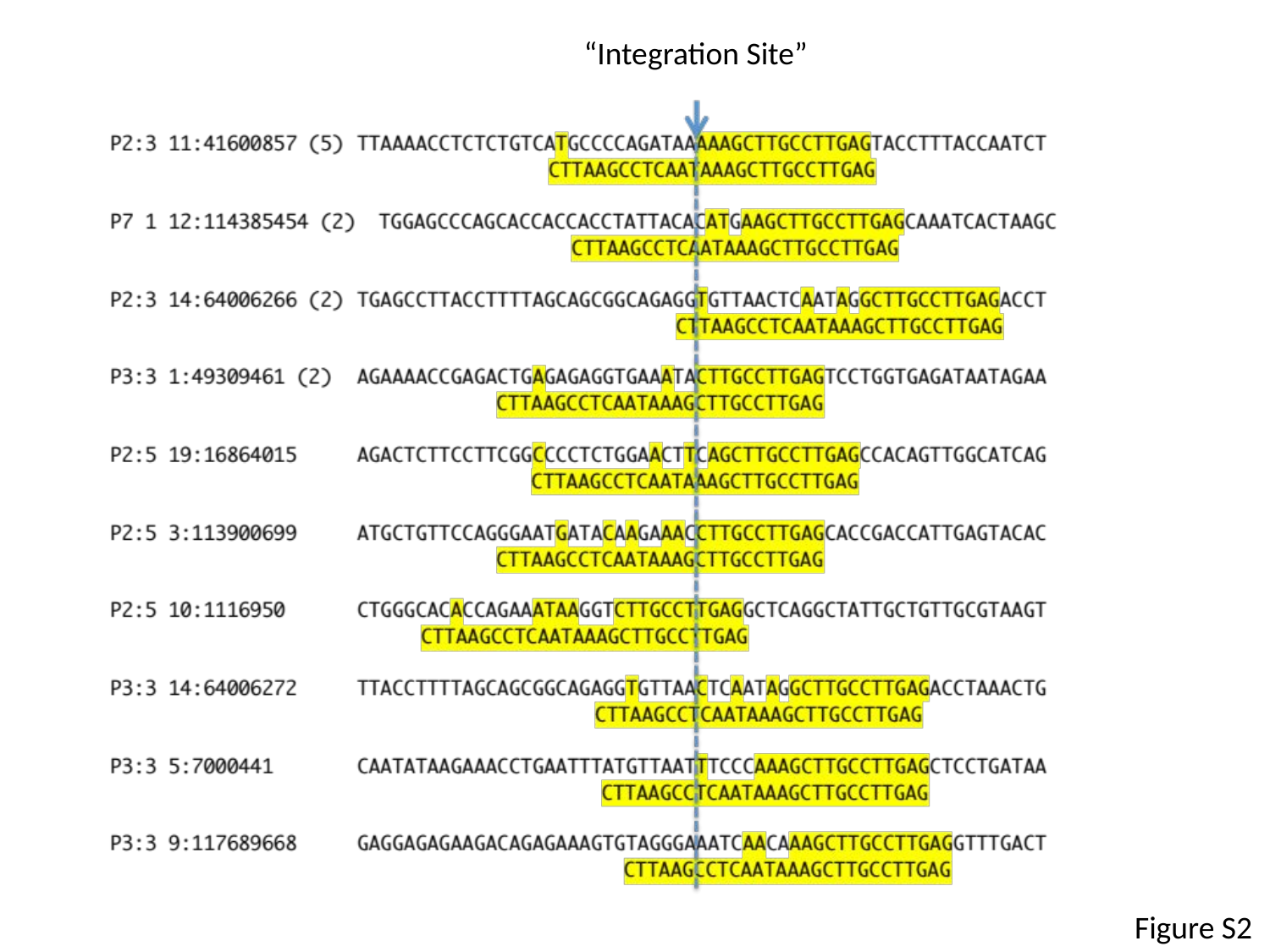

“Integration Site”
Figure S2

## Slide 3
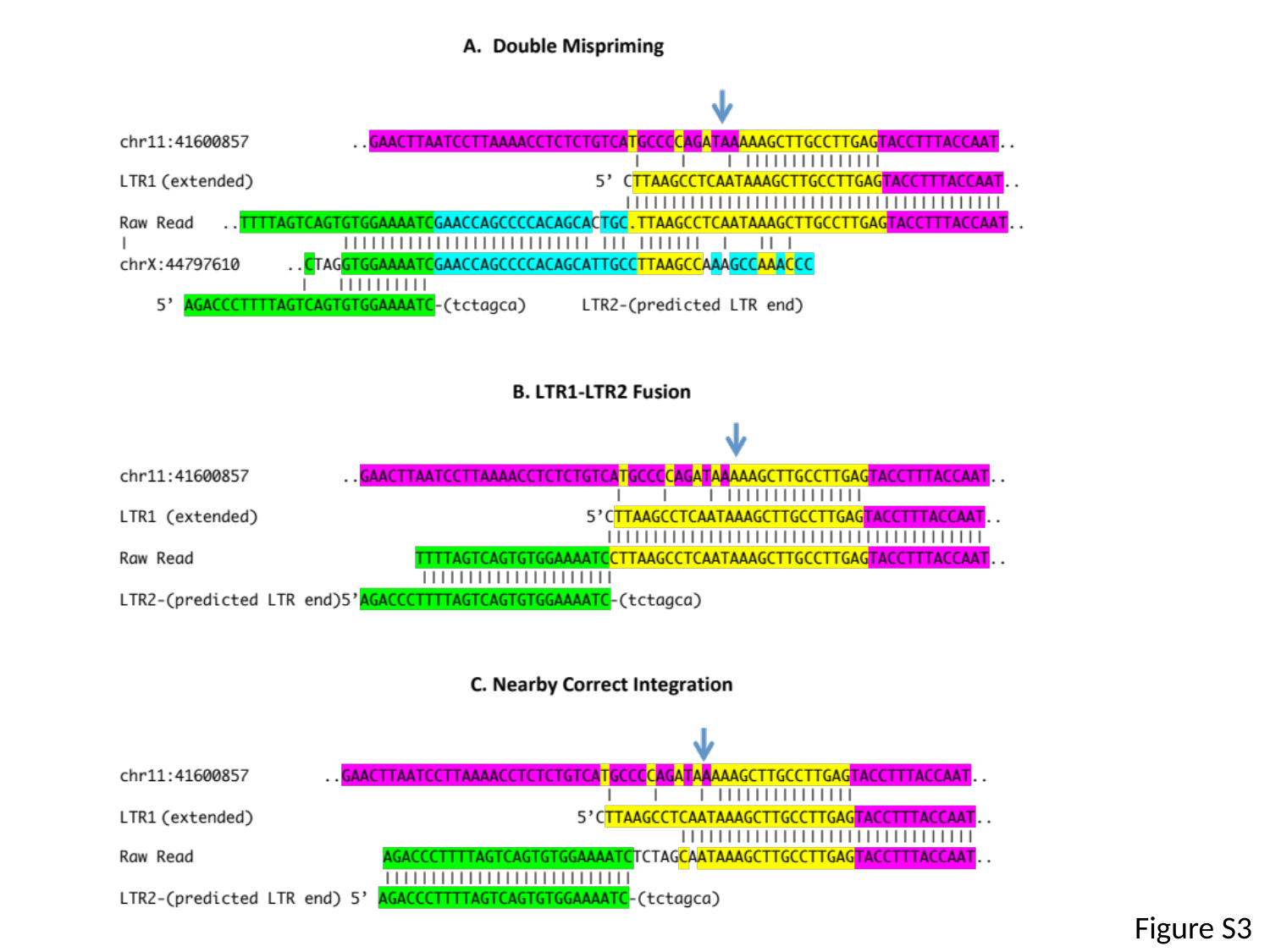

Figure S3

## Slide 4
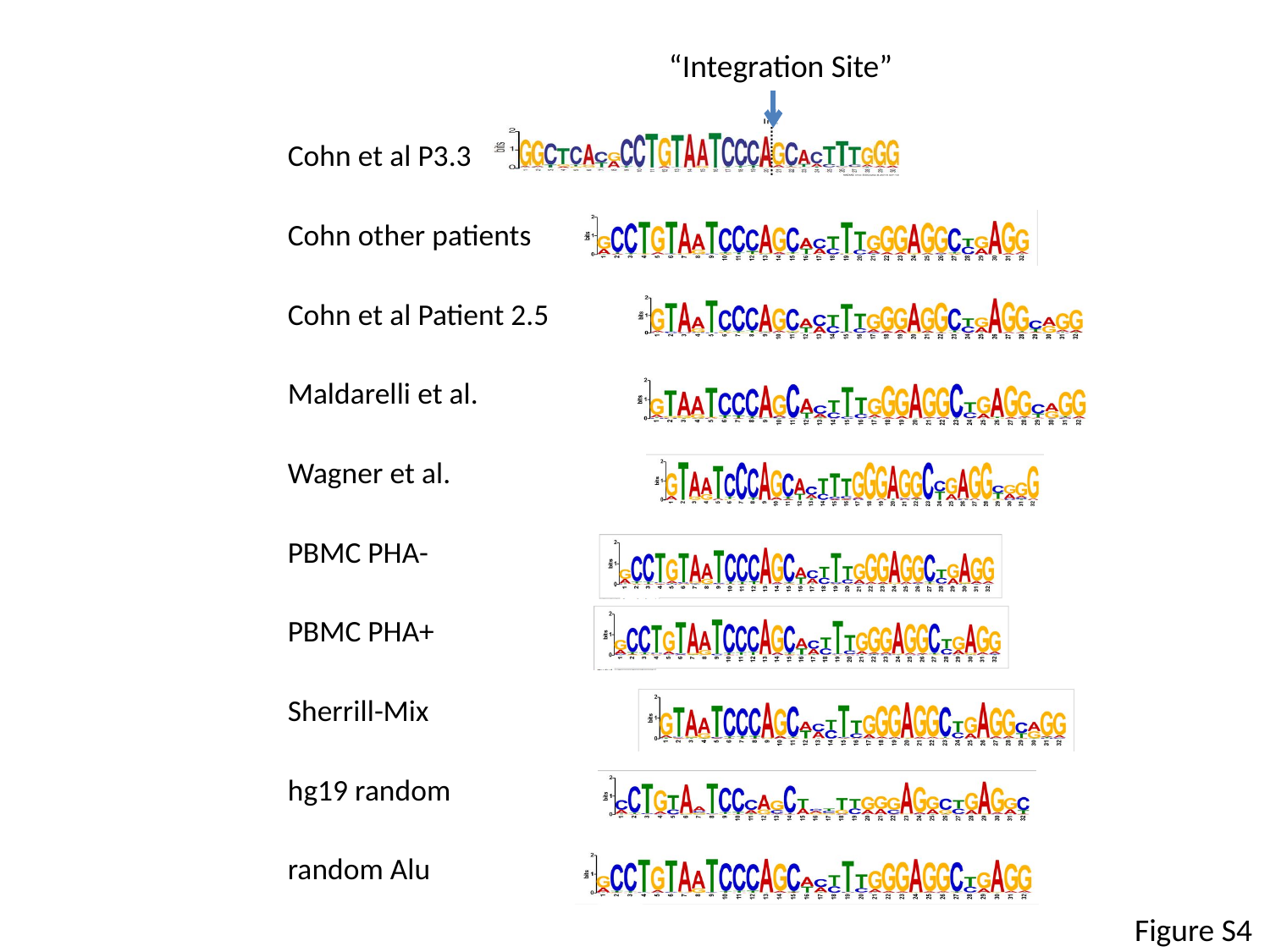

“Integration Site”
| |
| --- |
| |
| Cohn et al P3.3 |
| |
| Cohn other patients |
| |
| Cohn et al Patient 2.5 |
| |
| Maldarelli et al. |
| |
| Wagner et al. |
| |
| PBMC PHA- |
| |
| PBMC PHA+ |
| |
| Sherrill-Mix |
| |
| hg19 random |
| |
| random Alu |
| |
| |
| |
Figure S4

## Slide 5
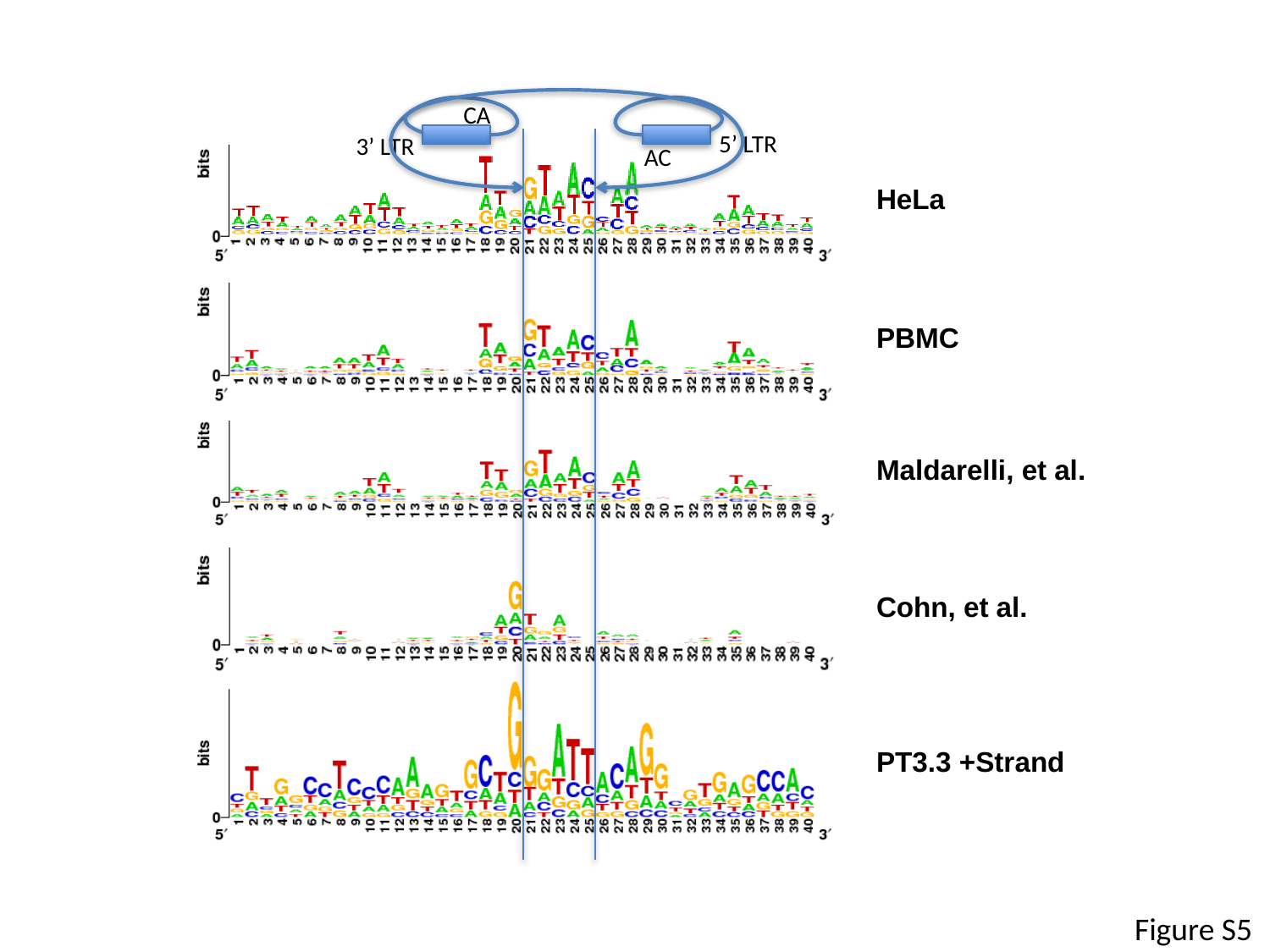

CA
5’ LTR
3’ LTR
AC
HeLa
PBMC
Maldarelli, et al.
Cohn, et al.
PT3.3 +Strand
Figure S5

## Slide 6
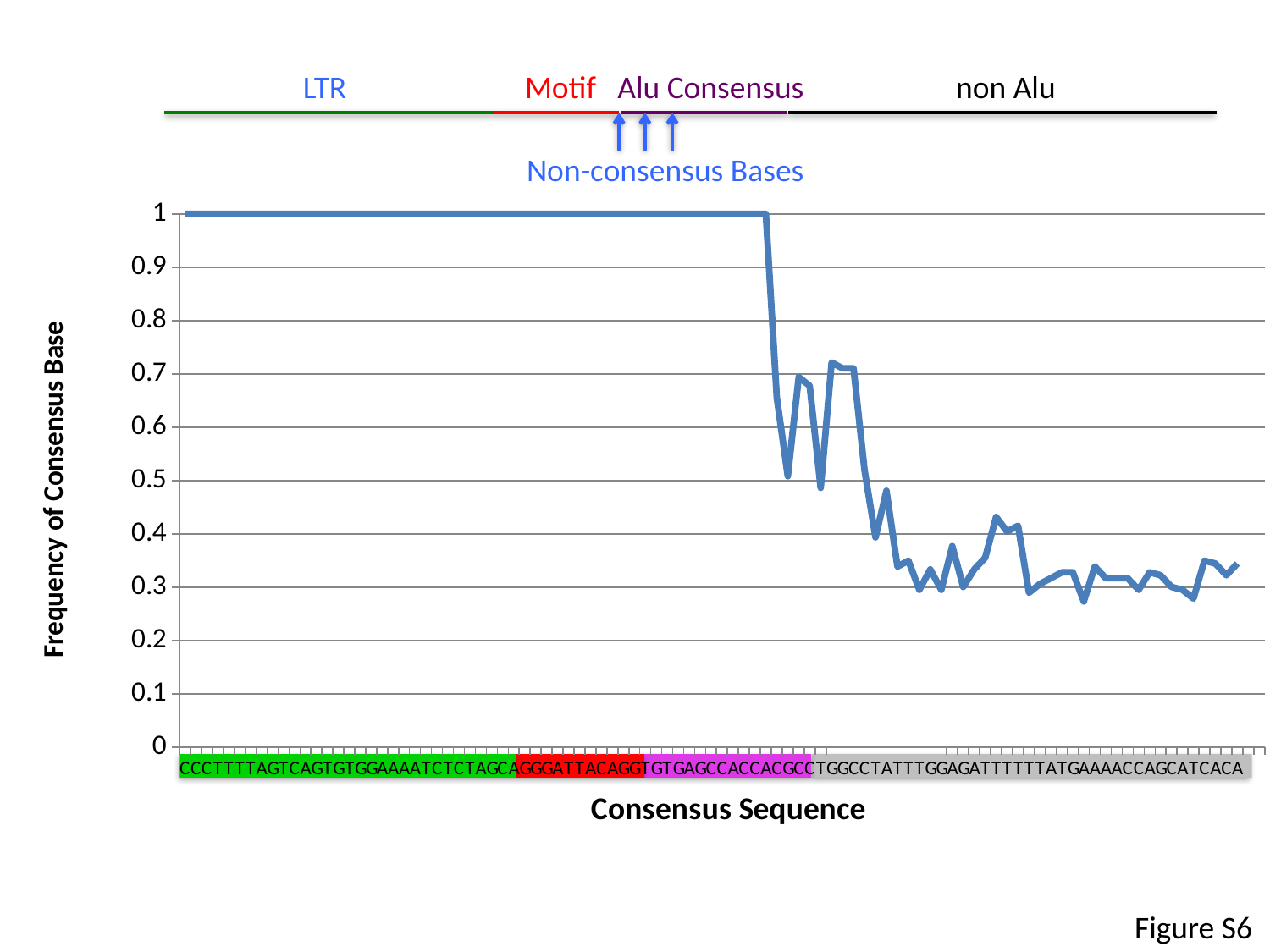

LTR
Motif
Alu Consensus
non Alu
Non-consensus Bases
### Chart
| Category | |
|---|---|
| C | 1.0 |
| C | 1.0 |
| C | 1.0 |
| T | 1.0 |
| T | 1.0 |
| T | 1.0 |
| T | 1.0 |
| A | 1.0 |
| G | 1.0 |
| T | 1.0 |
| C | 1.0 |
| A | 1.0 |
| G | 1.0 |
| T | 1.0 |
| G | 1.0 |
| T | 1.0 |
| G | 1.0 |
| G | 1.0 |
| A | 1.0 |
| A | 1.0 |
| A | 1.0 |
| A | 1.0 |
| T | 1.0 |
| C | 1.0 |
| T | 1.0 |
| C | 1.0 |
| T | 1.0 |
| A | 1.0 |
| G | 1.0 |
| C | 1.0 |
| A | 1.0 |
| G | 1.0 |
| G | 1.0 |
| G | 1.0 |
| A | 1.0 |
| T | 1.0 |
| T | 1.0 |
| A | 1.0 |
| C | 1.0 |
| A | 1.0 |
| G | 1.0 |
| G | 1.0 |
| T | 1.0 |
| G | 1.0 |
| T | 1.0 |
| G | 1.0 |
| A | 1.0 |
| G | 1.0 |
| C | 1.0 |
| C | 1.0 |
| A | 1.0 |
| C | 1.0 |
| C | 1.0 |
| A | 1.0 |
| C | 0.655737704918033 |
| G | 0.508196721311475 |
| C | 0.693989071038251 |
| C | 0.6775956284153 |
| T | 0.486338797814208 |
| G | 0.721311475409836 |
| G | 0.710382513661202 |
| C | 0.710382513661202 |
| C | 0.519125683060109 |
| T | 0.39344262295082 |
| A | 0.480874316939891 |
| T | 0.33879781420765 |
| T | 0.349726775956284 |
| T | 0.295081967213115 |
| G | 0.333333333333333 |
| G | 0.295081967213115 |
| A | 0.377049180327869 |
| G | 0.300546448087432 |
| A | 0.333333333333333 |
| T | 0.355191256830601 |
| T | 0.431693989071038 |
| T | 0.404371584699454 |
| T | 0.415300546448087 |
| T | 0.289617486338798 |
| T | 0.306010928961749 |
| A | 0.316939890710382 |
| T | 0.327868852459016 |
| G | 0.327868852459016 |
| A | 0.273224043715847 |
| A | 0.33879781420765 |
| A | 0.316939890710382 |
| A | 0.316939890710382 |
| C | 0.316939890710382 |
| C | 0.295081967213115 |
| A | 0.327868852459016 |
| G | 0.322404371584699 |
| C | 0.300546448087432 |
| A | 0.295081967213115 |
| T | 0.278688524590164 |
| C | 0.349726775956284 |
| A | 0.344262295081967 |
| C | 0.322404371584699 |
| A | 0.344262295081967 |
Figure S6
